# Vitamin D receptor (VDR) gene polymorphisms and risk for polycystic ovary syndrome and infertility: An updated systematic review and meta-analysis

**DOI:** 10.1016/j.metop.2024.100343

**Published:** 2024-12-31

**Authors:** Roozbeh Heidarzadehpilehrood, Habibah Abdul Hamid, Maryam Pirhoushiaran

**Affiliations:** a Department of Obstetrics & Gynaecology, Medicine and Health Sciences, Universiti Putra Malaysia Serdang, Selangor, 43400, Malaysia; b Department of Medical Genetics, School of Medicine, Tehran University of Medical Sciences, 1417613151, Tehran, Iran

**Keywords:** Polycystic ovary syndrome, Single nucleotide polymorphism, Vitamin D receptor, *Apa*I, *Bsm*I, Cdx2

## Abstract

**Background:**

Vitamin D receptor (VDR) gene polymorphisms have been implicated in polycystic ovary syndrome (PCOS). Despite VDR gene polymorphisms importance and their risk for PCOS, they have not been extensively studied. The main objective was to evaluate the associations between VDR gene polymorphisms and risk for PCOS.

**Methods:**

The current systematic review and meta-analysis examined VDR gene polymorphisms with PCOS in case-control and cohort studies. Relevant keywords were used to search Scopus, Web of Science, PubMed, MEDLINE, ScienceDirect, and Google Scholar for peer-reviewed publications until July 1, 2024. Selected papers were assessed for risk bias and quality using the Modified Newcastle-Ottawa scale. A meta-analysis was conducted using a random-effect model. The association between VDR gene polymorphism(s) and PCOS in women was reported as odds ratios (ORs) with 95 % confidence intervals (CIs).

**Results:**

Twenty eligible studies, including 5618 subjects, were included in systematic review and meta-analysis. This study revealed a significant association between *Apa*I (rs7975232; OR = 1.18, 95 % CI = 1.06–1.30, *p < 0.01*), *Bsm*I (rs1544410; OR = 1.22, 95 % CI = 1.08–1.37, *p < 0.01),* Cdx2 (rs11568820; OR = 1.15, 95 % CI = 0.97–1.38, *p < 0.01*), and *Taq*I (rs731236; OR = 1.25, 95 % CI = 1.13–1.39, *p < 0.01*). However, there was no significant association in the *Fok*I (rs22228570; OR = 1.01, 95 % CI = 0.91–1.112, *p = 0.12*) polymorphism with PCOS risk.

**Conclusions:**

The present systematic review and meta-analysis shows that women with *Apa*I, *Bsm*I, Cdx2, and *Taq*I VDR gene polymorphisms may have a higher risk of PCOS. This study was registered on the Prospective International Registry of Systematic Reviews (PROSPERO) with registration number CRD42024564851.

## 1 Introduction

PCOS is an intricate condition with diverse effects on the reproductive, endocrine, metabolic, and cardiovascular systems [1]. Global estimates suggest that the condition affects between 5 % and 20 % of women throughout their reproductive years. PCOS development is influenced by genetic, epigenetic, and ethnic factors [2]. Women with PCOS have an impaired metabolite included hyperandrogenism, glucose tolerance, insulin resistance, hyperinsulinemia, and infertility due to unovulation [3]. There is increasing evidence that genetic and epigenetic factors are believed to play critical role in PCOS progression [[4], [5], [6]]. However, despite much research over the years, the exact underlying pathological mechanisms of PCOS remain uncertain.

VDR, a ligand-dependent transcription factor belonging to the steroid/thyroid hormone receptor superfamily, is responsible for vitamin D's effect [7]. Vitamin D regulates the female reproductive system through steroidogenesis and corresponding hormones: anti-mullerian hormone (AMH), follicle-stimulating hormone, progesterone in granulosa cells (GCs) and glucose hemostasis in pancreatic β-cells [8]. The VDR gene is located on chromosome 12q13.11 and encompasses 14 exons that encode a 427-amino-acid protein [9]. The VDR gene features five commonly reported single nucleotide polymorphisms (SNPs), notably *Apa*I (rs7975232) located in intron 8, *Bsm*I (rs1544410) located in intron 8, Cdx2 (rs11568820) located in exon 1, *Fok*I (rs10735810) located in exon 2, and *Taq*I (rs731236) located in exon 9 [9].

Understanding the associations between VDR gene polymorphisms and the risk of developing PCOS and infertility is critical for improving the diagnosis, treatment, and management of this condition. The controversial evidence shows an association between VDR gene polymorphisms and the risk of PCOS and infertility [10]. However, the relationship between the VDR gene polymorphisms and PCOS remained unclear. Previous meta-analyses have identified several VDR gene polymorphisms that play a role in the development of PCOS, including *Apa*I, *Bsm*I, *Fok*I [11]. The current systematic review and meta-analysis's main objective was to evaluate the associations between VDR gene polymorphisms and PCOS risk. A total of 5618 participants, focusing on five key VDR gene polymorphisms: *Apa*I (rs7975232), *Bsm*I (rs1544410), Cdx2 (rs11568820), *Fok*I (rs22228570), and *Taq*I (rs731236), were selected to evaluate risk associations for PCOS development. An updated meta-analysis that included more population studies may address research gaps in VDR gene polymorphisms as well as the risk for PCOS. We hypothesize that VDR gene polymorphisms are associated with an increased risk of PCOS in women.

## 2 Methods

The current systematic review and meta-analysis were conducted in accordance with the recommendations established by the preferred reporting items for systematic reviews and meta-analyses (PRISMA 2020) [12]. This systematic review and meta-analysis were registered on the PROSPERO with registration number CRD42024564851. The review protocol for this study is accessible at (https://www.crd.york.ac.uk/prospero/).

### 2.1 Eligibility criteria

Inclusion criteria included: (i) observational studies, case-control studies, and cohort studies; (ii) women diagnosed with PCOS; (iii) investigations to determine VDR gene polymorphisms *Apa*I (rs7975232), *Bsm*I (rs1544410), Cdx2 (rs11568820), *Fok*I (rs2228570), and *Taq*I (rs731236). (iv) Studies that have reported the frequencies of genotypes or alleles by comparing at least two groups, a group including PCOS or cases against non-PCOS or healthy or control groups; (v) Studies that report ORs and 95 % CIs. Exclusion criteria included: (i) studies without a clear diagnosis of PCOS; (ii) studies not reporting on VDR gene polymorphisms; (iii) all other types of studies, clinical trial studies, reviews, meta-analyses, cross-sectional studies, case reports, case series, commentaries, letters to the editor, and reports; (iv) case-control studies without reporting inclusion criteria; (v) repetitive and non-English language studies; (vi) studies without reporting ORs and 95 % CIs.

### 2.2 Methodology for searching and screening

We employed a number of worldwide databases, for instance, Scopus, Web of Science, PubMed, MEDLINE, and Google Scholar, to perform the meta-analysis. These databases were searched for papers published up to July 2024, using specific search keywords and their synonyms: "vitamin D receptor," or "VDR," and "polycystic ovary syndrome," or "PCOS," and "SNP," or "polymorphism." In addition, we manually searched these databases, closely checking the references to pertinent research and scanning the published literature for any other related studies that might not have come up in the first database search. Two independent writers (R.H., M.P.) separately conducted the screening process (abstracts and full text using eligibility criteria) to guarantee correctness and thoroughness.

### 2.3 Data extraction and management

Data were obtained using a standardized form and were separately collected by two reviewers from the chosen studies. Corresponding authors were contacted as necessary to provide clarification or validate data. We investigate the association between VDR gene polymorphisms (*Apa*I, *Bsm*I, Cdx2, *Fok*I, and *Taq*I) and the susceptibility to PCOS in women. This data covered a number of important variables, including the study's site, authors, year of publication, sample size, distinctive characteristics, population characteristics, age distribution, research site, participant ethnicity, and PCOS criteria. Details about the genotyping techniques utilized, the number of cases and controls related to the VDR gene polymorphisms, the outcome measured, and the published results are all included. It was considered that missing data would not be reported if the authors could not be reached for clarification.

### 2.4 Bias risk

The methodological quality and risk assessment of non-randomized studies in meta-analyses was conducted using the Modified Newcastle-Ottawa Quality Assessment Scale checklist. There are eight components total, broken down into three categories: ascertainment of exposure, comparability of cases and controls, and selection of cases and controls. An item may receive a maximum of one star for each of the first two categories and a maximum of two stars for comparability. There was a 0–9 point scoring system. A score of six or higher indicates the excellent quality of a study [13]. Two reviewers (R.H. and M.P.) independently conducted the risk of bias evaluation. In cases of inconsistency, a third author (H.A.B.) was consulted, and discrepancies were resolved through consensus. No automation tools were used during this process.

### 2.5 Statistical analysis

The Hardy–Weinberg equilibrium (HWE) was used to evaluate the control genotype distribution (*p* < 0.05 was deemed relevant). The influence on a forest plot and the intensity of the association between VDR polymorphisms and the risk of PCOS were estimated using the computation of ORs and 95 % CIs in six distinct genetic models. *Apa*I (rs7975232) allele contrast (A vs. a), recessive model (AA vs. Aa + aa), dominant model (AA + Aa vs. aa), over dominant model (Aa vs. AA + aa), AA vs. aa model, and AA vs. Aa model; *Bsm*I (rs1544410) allele contrast (B vs. b), recessive model (BB vs. Bb + bb), dominant model (BB + Bb vs. bb), over dominant model (Bb vs. BB + bb), BB vs. bb model, and BB vs. Bb model. Cdx2 (rs11568820) allele contrast (C vs. c), recessive model (CC vs. *Cc* + cc), dominant model (CC + *Cc* vs. cc), over-dominant model (*Cc* vs. CC + cc), CC vs. cc model, and CC vs. *Cc* model. *Fok*I (rs2228570) allele contrast (F vs. f), recessive model (FF vs. Ff + ff), dominant model (FF + Ff vs. ff), over-dominant model (Ff vs. FF + ff), FF vs. ff model, FF vs. Ff model, and Ff vs. ff model; *Taq*I (rs731236) allele contrast (T vs. t), recessive model (TT vs. Tt + tt), dominant model (TT + Tt vs. tt), over dominant model (Tt vs. TT + tt), TT vs. tt model, and TT vs. Tt model; Also, *I*^*2*^ and Q Cochrane tests were used to evaluate the heterogeneity throughout the studies. Egger's test (*p* < 0.05) and funnel plots were utilized to test for publication biases. MetaGenyo, an online tool for doing a meta-analysis of genetic association research, was used for all statistical analyses [14].

## 3 Results

### 3.1 Characteristics of eligible studies

**Fig. 1:**
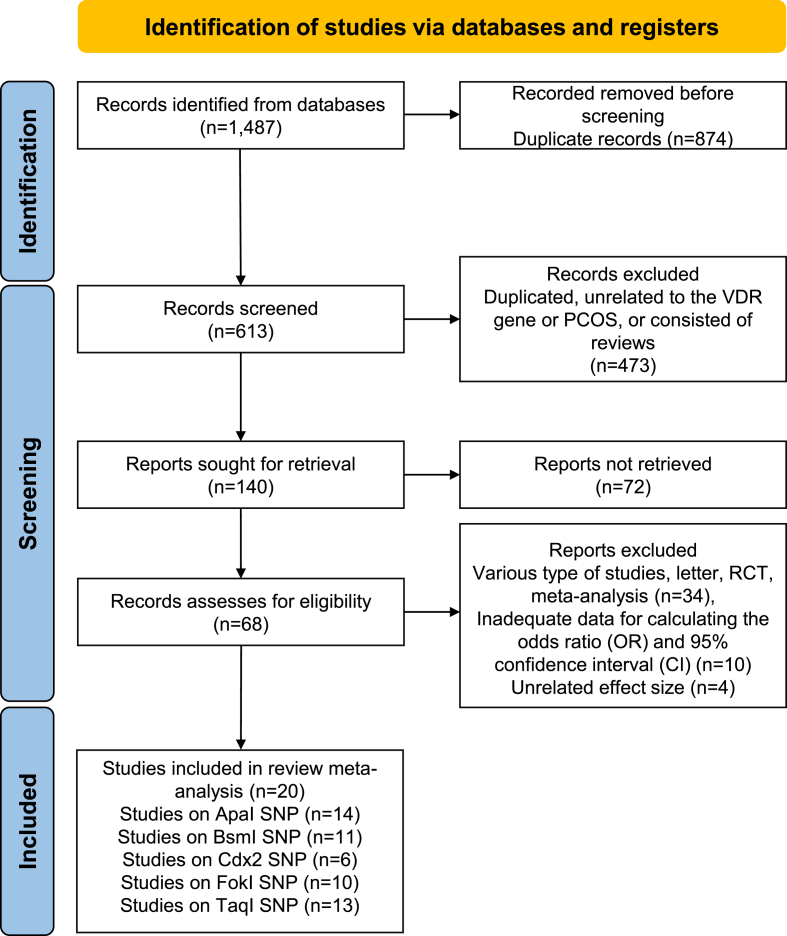
PRISMA 2020 flow diagram of included studies in meta-analysis.

Fig. 1 illustrates the study selection method for this meta-analysis, which adhered to the PRISMA flow diagram. Initially, we conducted a comprehensive search of 1487 records from the aforementioned databases. We excluded a total of 874 records from the search results due to their duplication. After screening the remaining 613 records, we excluded 473 records because they were duplicates, unrelated to the VDR gene or PCOS, or consisted of reviews. 140 publications were sought to be retrieved, of which 72 records were not retrieved. The remaining 68 publications were downloaded, subjected to a meticulous, complete publishing evaluation and assessed for eligibility. We excluded 48 studies due to various types of studies, letters, RCTs, and meta-analyses, as well as their lack of a case-control design and inadequate data for calculating the ORs and 95 % CI. In conclusion, a total of 20 papers [9,10,[15], [16], [17], [18], [19], [20], [21], [22], [23], [24], [25], [26], [27], [28], [29], [30], [31], [32]] were included in this systematic review and meta-analysis. Notably, the objective criteria, including the PCOS diagnostic criteria, healthy control groups, mean or age of study group, the Newcastle-Ottawa scale (NOS) for assessing the quality of nonrandomized studies, genotyping techniques that were employed, study sets, the ethnicity of the population that was included in the meta-analysis, and the total cases and controls for each study. The included studies provide the genotype and allele frequencies, in addition to other features (Table 1, Table 2, Table 3, Table 4).

**Table 1:** Presents the characteristics of studies that examined VDR gene polymorphisms in individuals with polycystic ovarian syndrome compared to control groups.

Author	Year	Country	Ethnicity	Diagnostic criteria	Genotyping methods	Age PCOS/Control (Mean)	PCOS (n)	Control (n)
*Apa*I (rs7975232)								
Albahlol et al.	2023	Egypt	Caucasian	Rotterdam	real-time PCR,	24.09 ± 3.84/21.57 ± 3.63	185	207
Szafarowska et al.	2019	Poland	Caucasian	Rotterdam	real-time PCR,	33.9 (4.0)	75	23
Santos et al.	2018	Brazil	Caucasian	Rotterdam	real-time PCR,	22.89 ± 6.66/25.18 ± 7.72	191	100
Humadi et al.	2018	Iraq	Asian	Rotterdam	real-time PCR,	N/A	100	100
Al Thomal et al.	2018	Saudi Arabia	Asian	Rotterdam	real-time PCR,	18–36	17	17
Siddamalla et al.	2017	India	Asian	Rotterdam	PCR-RFLP	N/A	95	130
Abdul-hassan et al.	2017	Iraq	Asian	Rotterdam	real-time PCR,	15–45	50	50
Cao et al.	2016	China	Asian	Rotterdam	PCR-RFLP	28.92 (0.41)	120	120
Dasgupta et al.	2015	India	Asian	Rotterdam	PCR-RFLP	N/A	250	250
Jedrzejuk et al.	2015	Poland	Caucasian	Rotterdam	PCR and minisequencing	20–35	90	98
Mahmoudi et al.	2015	Iran	Asian	Rotterdam	PCR-RFLP	28.43/30.29	35	35
El-Shal et al.	2013	Egypt	Caucasian	Rotterdam	PCR-RFLP	29.8 ± 5.6/29.3 ± 6.2	150	150
Wehr et al.	2011	Australia	Caucasian	Rotterdam	NucleoSpin Blood method	16–45	543	145
Mahmoudi et al.	2009	Iran	Asian	Rotterdam	PCR	19–42	162	162
*Bsm*I (rs1544410)								
Albahlol et al.	2023	Egypt	Caucasian	Rotterdam	Real-time PCR,	24.09 ± 3.84/21.57 ± 3.63	185	207
Ramezani et al.	2020	Iran	Asian	Rotterdam	Real-time PCR,	18–40	38	40
Szafarowska et al.	2019	Poland	Caucasian	Rotterdam	Real-time PCR,	33.9 (4.0)	75	23
Santos et al.	2018	Brazil	Caucasian	Rotterdam	Real-time PCR,	22.89 ± 6.66 25.18 ± 7.72	191	100
Siddamalla et al.	2017	India	Asian	Rotterdam	PCR-RFLP	N/A	95	130
Cao et al.	2016	China	Asian	Rotterdam	PCR-RFLP	28.92 (0.41)	120	120
Jedrzejuk et al.	2015	Poland	Caucasian	Rotterdam	PCR and minisequencing	20–35	173	125
Mahmoudi et al.	2015	Iran	Asian	Rotterdam	Real-time PCR,	28.43/30.29	35	35
Bagheri et al.	2012	Iran	Asian	Rotterdam	PCR-RFLP	26.58 ± 3.33/28.24 ± 5.25	46	46
Wehr et al.	2011	Australia	Caucasian	Rotterdam	NucleoSpin Blood method	16–45	543	145
Mahmoudi et al.	2009	Iran	Asian	Rotterdam	PCR	19–42	162	162
Cdx2 (rs11568820)								
Khansari et al.	2023	Iran	Asian	N/A	ASM-PCR	28.58 ± 5.83/34.37 ± 5.07	48	38
Albahlol et al.	2023	Egypt	Caucasian	Rotterdam	Real-time PCR,	24.09 ± 3.84/21.57 ± 3.63	185	207
Szafarowska et al.	2019	Poland	Caucasian	Rotterdam	Real-time PCR,	33.9 (4.0)	75	23
Malik et al.	2018	Pakistan	Asian	Rotterdam	T-ARMS-PCR	N/A	186	91
Dasgupta et al.	2015	India	Asian	Rotterdam	PCR-RFLP	N/A	250	250
Wehr et al.	2011	Australia	Caucasian	Rotterdam	NucleoSpin Blood method	16–45	543	145
*Fok*I (rs2228570)								
Song et al.	2019	South Korea	Asian	Rotterdam	–	24 ± 5/27 ± 5	432	927
Al Thomal et al.	2018	Saudi Arabia	Asian	Rotterdam	real-time PCR,	18–36	17	17
Abdul-hassan et al.	2017	Iraq	Asian	Rotterdam	real-time PCR,	15–45	50	50
Cao et al.	2016	China	Asian	Rotterdam	PCR-RFLP	28.92 (0.41)	120	120
Dasgupta et al.	2015	India	Asian	Rotterdam	PCR-RFLP	N/A	250	250
Jedrzejuk et al.	2015	Poland	Caucasian	Rotterdam	PCR and minisequencing	20–35	173	125
Mahmoudi et al.	2015	Iran	Asian	Rotterdam	PCR-RFLP	28.43/30.29	35	35
Bagheri et al.	2012	Iran	Asian	Rotterdam	PCR-RFLP	26.58 ± 3.33/28.24 ± 5.25	46	46
Wehr et al.	2011	Australia	Caucasian	Rotterdam	NucleoSpin Blood method	16–45	543	145
Mahmoudi et al.	2009	Iran	Asian	Rotterdam	PCR	19–42	162	162
*Taq*I (rs731236)								
Albahlol et al.	2023	Egypt	Caucasian	Rotterdam	real-time PCR,	24.09 ± 3.84/21.57 ± 3.63	185	207
Santos et al.	2018	Brazil	Caucasian	Rotterdam	real-time PCR,	22.89 ± 6.66 25.18 ± 7.72	191	100
Al Thomal et al.	2018	Saudi Arabia	Asian	Rotterdam	real-time PCR,	18–36	17	17
Siddamalla et al.	2017	India	Asian	Rotterdam	PCR-RFLP	N/A	95	130
Abdul-hassan et al.	2017	Iraq	Asian	Rotterdam	real-time PCR,	15–45	50	50
Cao et al.	2016	China	Asian	Rotterdam	PCR-RFLP	28.92 (0.41)	120	120
Dasgupta et al.	2015	India	Asian	Rotterdam	PCR-RFLP	N/A	250	250
Jedrzejuk et al.	2015	Poland	Caucasian	Rotterdam	PCR and minisequencing	20–35	173	125
Mahmoudi et al.	2015	Iran	Asian	Rotterdam	PCR-RFLP	28.43/30.29	35	35
El-Shal et al.	2013	Egypt	Caucasian	Rotterdam	PCR-RFLP	29.8 ± 5.6/29.3 ± 6.2	150	150
Bagheri et al.	2013	Iran	Asian	Rotterdam	PCR-RFLP	26.03 ± 4.98/27.18 ± 4.95	38	38
Wehr et al.	2011	Australia	Caucasian	Rotterdam	NucleoSpin Blood method	16–45	543	145
Mahmoudi et al.	2009	Iran	Asian	Rotterdam	PCR	19–42	162	162

**Table 2:** Key findings from the meta-analysis of VDR gene polymorphisms in polycystic ovarian syndrome using pooled ORs.

Genetic models		Study	Number of studies	Test of association				Test of heterogeneity		Publication bias
				OR	95 % CI	*p*	Model	*p*	I^2	*p* (Egger's test)
*Apa*I (rs7975232)										
Allele contrast	A vs. a	Overall	14	1.176	[1.0626; 1.3003]	0.002	Fixed	0.007	0.547	0.405
Recessive model	AA vs. Aa + aa	Overall	14	1.268	[1.0493; 1.5315]	0.014	Fixed	0.001	0.630	0.573
Dominant model	AA + Aa vs. aa	Overall	14	1.207	[1.0468; 1.3919]	0.010	Fixed	0.167	0.268	0.465
Overdominant	Aa vs. AA + aa	Overall	14	1.031	[0.8938; 1.1892]	0.675	Fixed	0.007	0.546	0.678
Pairw1	AA vs. aa	Overall	14	1.385	[1.1231; 1.7081]	0.002	Fixed	0.008	0.540	0.360
Pairw2	AA vs. Aa	Overall	14	1.238	[1.0028; 1.5271]	0.047	Fixed	0.001	0.641	0.354
*Bsm*I (rs1544410)										
Allele contrast	B vs. b	Overall	11	1.501	[1.3303; 1.6944]	0.000	Fixed	0.000	0.777	0.228
Recessive model	BB vs. Bb + bb	Overall	11	1.664	[1.3454; 2.0582]	0.000	Fixed	0.000	0.690	0.548
Dominant model	BB + Bb vs. bb	Overall	11	1.667	[1.3853; 2.0067]	0.000	Fixed	0.000	0.707	0.042
Overdominant	Bb vs. BB + bb	Overall	11	1.016	[0.8604; 1.1994]	0.853	Fixed	0.037	0.480	0.450
Pairw1	BB vs. bb	Overall	11	2.265	[1.7386; 2.9509]	0.000	Fixed	0.000	0.725	0.481
Pairw2	BB vs. Bb	Overall	11	1.491	[1.1906; 1.8665]	0.001	Fixed	0.009	0.578	0.390
Cdx2 (rs11568820)										
Allele contrast	C vs. c	Overall	6	1.155	[0.9695; 1.3757]	0.107	Fixed	0.000	0.959	0.841
Recessive model	CC vs. *Cc* + cc	Overall	6	3.464	[2.0442; 5.8713]	0.000	Fixed	0.002	0.765	0.722
Dominant model	CC + *Cc* vs. cc	Overall	6	0.935	[0.7568; 1.1554]	0.534	Fixed	0.000	0.959	0.969
Overdominant	*Cc* vs. CC + cc	Overall	6	0.690	[0.5545; 0.8595]	0.001	Fixed	0.000	0.933	0.968
Pairw1	CC vs. cc	Overall	6	4.008	[2.3413; 6.8610]	0.000	Fixed	0.000	0.833	0.543
Pairw2	CC vs. *Cc*	Overall	6	2.920	[1.6397; 5.2013]	0.000	Fixed	0.101	0.484	0.794
*Fok*I (rs2228570)										
Allele contrast	F vs. f	Overall	10	1.008	[0.9062; 1.1210]	0.885	Fixed	0.124	0.355	0.159
Recessive model	FF vs. Ff + ff	Overall	10	0.904	[0.7223; 1.1319]	0.379	Fixed	0.474	0.000	0.231
Dominant model	FF + Ff vs. ff	Overall	10	1.058	[0.9155; 1.2237]	0.443	Fixed	0.289	0.168	0.133
Overdominant	Ff vs. FF + ff	Overall	10	1.101	[0.9545; 1.2696]	0.187	Fixed	0.768	0.000	0.071
Pairw1	FF vs. ff	Overall	10	0.939	[0.7357; 1.1979]	0.612	Fixed	0.322	0.132	0.211
Pairw2	FF vs. Ff	Overall	10	0.885	[0.6984; 1.1219]	0.313	Fixed	0.743	0.000	0.348
*Taq*I (rs731236)										
Allele contrast	T vs. t	Overall	13	1.2221	[1.0996; 1.3582]	0.000	Fixed	0.000	0.804	0.723
Recessive model	TT vs. Tt + tt	Overall	13	1.467	[1.1845; 1.8169]	0.000	Fixed	0.001	0.629	0.728
Dominant model	TT + Tt vs. tt	Overall	13	1.1968	[1.0346; 1.3845]	0.016	Fixed	0.000	0.729	0.957
Over dominant	Tt vs. TT + tt	Overall	13	0.9706	[0.8392; 1.1225]	0.687	Fixed	0.088	0.370	0.756
Pairw1	TT vs. tt	Overall	13	1.5235	[1.2108; 1.9169]	0.000	Fixed	0.000	0.695	0.784
Pairw2	TT vs. Tt	Overall	13	1.3976	[1.1117; 1.7570]	0.004	Fixed	0.046	0.437	0.805

**Table 3:** Genotype and allele distribution of VDR gene polymorphisms in polycystic ovary syndrome.

Study	Year	Ethnicity	PCOS					Control					HW *p*	HW *adj-p*
*Apa*I (rs7975232)			a	A	aa	Aa	AA	a	A	aa	Aa	AA		
Albahlol et al.	2023	Egyptian	118	252	32	54	99	88	326	14	60	133	0.0536	0.1501
Szafarowska et al.	2019	Polish	41	109	3	35	37	11	35	1	9	13	0.7179	0.9137
Santos et al.	2018	Brazilian	170	210	41	88	61	80	120	16	48	36	1	1
Humadi et al.	2018	Iraqi	56	144	16	24	60	58	82	28	2	40	0	0
Al Thomal et al.	2018	Saudi	9	23	1	7	8	10	22	1	8	7	0.5128	0.7977
Siddamalla et al.	2017	Indian	85	105	32	21	42	85	175	25	35	70	0	0
Abdul-hassan et al.	2017	Iraqi	30	70	1	28	21	35	65	6	23	21	0.9381	1
Cao et al.	2016	Han Chinese	138	102	40	58	22	107	133	26	55	39	0.4274	0.748
Dasgupta et al.	2015	Indian	146	354	13	120	117	143	357	13	117	120	0.0211	0.0985
Jedrzejuk et al.	2015	Polish	90	90	19	52	19	83	113	17	49	32	0.8123	0.9477
Mahmoudi et al.	2015	Iranian	29	41	9	11	15	33	37	6	21	8	0.2276	0.4552
El-Shal et al.	2013	Egyptian	109	191	22	65	63	100	200	18	64	68	0.6242	0.8739
Wehr et al.	2011	Australian	528	558	127	274	142	134	156	37	60	48	0.0435	0.1501
Mahmoudi et al.	2009	Iranian	140	184	36	68	58	136	188	23	90	49	0.0738	0.1722

Study	Year	Ethnicity	PCOS					Control					HW *p*	HW *adj-p*
*Bsm*I (rs1544410)			b	B	bb	Bb	BB	b	B	bb	Bb	BB		
Albahlol et al.	2023	Egyptian	111	259	30	51	104	353	61	7	47	153	0.1656	0.3643
Ramezani et al.	2020	Iranian	16	60	3	10	25	62	18	1	16	23	0.3527	0.6466
Szafarowska et al.	2019	Polish	98	52	31	36	8	19	27	8	11	4	0.9478	0.9772
Santos et al.	2018	Brazilian	150	224	37	76	74	130	70	11	48	41	0.5827	0.7228
Siddamalla et al.	2017	Indian	75	115	15	45	35	185	75	17	41	72	0.0082	0.0902
Cao et al.	2016	Han Chinese	134	106	37	60	23	135	105	25	55	40	0.4512	0.709
Jedrzejuk et al.	2015	Polish	107	73	31	45	14	68	128	43	42	13	0.5914	0.7228
Mahmoudi et al.	2015	Iranian	38	32	13	12	10	33	37	7	23	5	0.0595	0.2182
Bagheri et al.	2012	Iranian	35	57	4	27	15	64	28	2	24	20	0.1154	0.3174
Wehr et al.	2011	Australian	676	398	216	244	77	164	110	22	66	49	0.9772	0.9772
Mahmoudi et al.	2009	Iranian	191	133	53	85	24	127	197	53	91	18	0.0231	0.127

Study	Year	Ethnicity	PCOS					Control					HW *p*	HW *adj-p*
Cdx2 (rs11568820)			c	C	cc	*Cc*	CC	c	C	cc	*Cc*	CC		
Khansari et al.	2023	Iranian	6	70	0	6	32	5	75	0	5	35	0.6733	0.6733
Albahlol et al.	2023	Egyptian	173	197	51	71	63	61	353	8	45	154	0.0524	0.1048
Szaflarowska et al.	2019	Polish	30	120	7	16	52	14	32	1	12	10	0.2656	0.3984
Malik et al.	2018	Pakistani	24	136	7	10	63	30	126	9	12	57	0	0
Dasgupta et al.	2015	Indian	68	432	8	52	190	143	357	0	143	107	0	0
Wehr et al.	2011	Australian	222	868	23	176	346	52	238	3	46	96	0.3483	0.418

Study	Year	Ethnicity	PCOS					Control					HW *p*	HW *adj-p*
*Fok*I (rs2228570)			f	F	ff	Ff	FF	f	F	ff	Ff	FF		
Song et al.	2019	Korean	346	518	67	212	153	753	1101	159	435	333	0.4073	0.831
Al Thomali et al.	2018	Saudi	8	24	1	6	9	6	28	1	4	12	0.4322	0.831
Abdul-hassan et al.	2017	Iraqi	42	58	2	38	10	36	64	0	36	14	0.0001	0.001
Cao et al.	2016	Han Chinese	60	180	10	40	70	65	175	10	45	65	0.5798	0.831
Dasgupta et al.	2015	Indian	103	397	8	87	155	106	392	9	88	152	0.3882	0.831
Jedrzejuk et al.	2015	Polish	73	107	11	51	28	100	96	25	50	23	0.8366	0.9729
Mahmoudi et al.	2015	Iranian	21	49	2	17	16	12	58	1	10	24	0.9729	0.9729
Bagheri et al.	2012	Iranian	28	64	4	20	22	19	73	2	15	29	0.9727	0.9729
Wehr et al.	2011	Australian	405	671	82	241	215	104	166	22	60	53	0.4739	0.831
Mahmoudi et al.	2009	Iranian	91	233	12	67	83	73	251	7	59	96	0.5817	0.831

Study	Year	Ethnicity	PCOS					Control					HW *p*	HW *adj-p*
*Taq*I (rs731236)			t	T	tt	Tt	TT	t	T	tt	Tt	TT		
Albahlol et al.	2023	Egyptian	118	252	35	48	102	48	366	5	38	164	0.1327	0.345
Santos et al.	2018	Brazilian	149	227	31	87	70	70	128	11	48	40	0.5458	0.645
Al Thomali et al.	2018	Saudi	16	16	4	8	4	15	19	4	7	6	0.4965	0.645
Siddamalla et al.	2017	Indian	79	111	24	31	40	76	184	17	42	71	0.0125	0.0542
Abdul-hassan et al.	2017	Iraqi	41	59	1	39	10	49	51	6	37	7	0.0007	0.0091
Cao et al.	2016	Han Chinese	74	166	11	52	57	88	152	8	72	40	0.0014	0.0091
Dasgupta et al.	2015	Indian	186	318	47	92	113	181	325	38	105	110	0.1236	0.345
Jedrzejuk et al.	2015	Polish	61	119	8	45	37	61	135	12	37	49	0.2373	0.4436
Mahmoudi et al.	2015	Iranian	26	44	6	14	15	24	46	4	16	15	0.9317	0.9317
El-Shal et al.	2013	Egyptian	146	154	36	74	40	101	199	20	61	69	0.273	0.4436
Bagheri et al.	2013	Iranian	30	46	8	14	16	23	53	2	19	17	0.2552	0.4436
Wehr et al.	2011	Australian	382	690	72	238	226	111	163	23	65	49	0.8547	0.9259
Mahmoudi et al.	2009	Iranian	111	213	20	71	71	104	220	14	76	72	0.332	0.4796

**Table 4:** The Modified Newcastle-Ottawa Scale was used to assess the quality of selected studies.

Author	Year	Modified Newcastle–Ottawa scale			
		Selection	Comparability	Outcomes	Overall
Albahlol et al.	2023	4	1	2	7
Khansari et al.	2023	2	1	3	6
Ramezani et al.	2020	4	1	3	8
Szafarowska et al.	2019	4	1	3	8
Song et al.	2019	4	1	3	8
Santos et al.	2018	3	1	3	7
Humadi et al.	2018	3	1	3	7
Al Thomal et al.	2018	3	1	2	6
Malik et al.	2018	4	1	3	8
Siddamalla et al.	2017	4	0	3	7
Abdul-hassan et al.	2017	3	1	2	6
Cao et al.	2016	4	1	2	7
Dasgupta et al.	2015	4	0	3	7
Jedrzejuk et al.	2015	3	1	3	7
Mahmoudi et al.	2015	4	1	3	8
El-Shal et al.	2013	4	1	3	8
Bagheri et al.	2013	3	1	3	7
Bagheri et al.	2012	3	0	3	6
Wehr et al.	2011	4	1	3	8
Mahmoudi et al.	2009	3	1	3	7

### 3.2 Polymorphism *Apa*I (rs7975232)

**Fig. 2:**
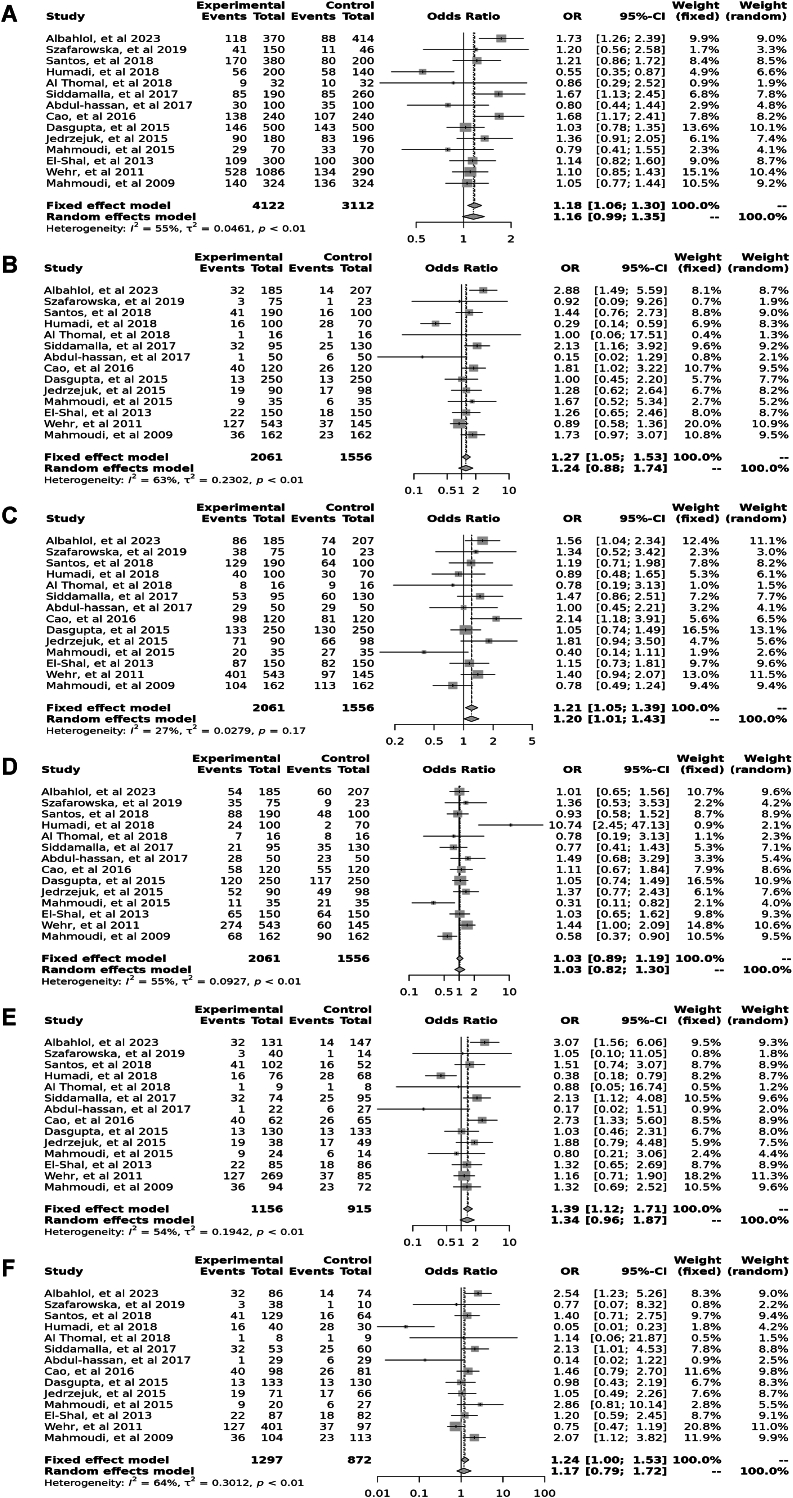
Forest plot illustrating the various genotypes and alleles in *Apa*I (rs7975232) in PCOS women vs. control groups. A: Allele contrast (A vs. a). B: Recessive model (AA vs. Aa + aa). C: Dominant model (AA + Aa vs. aa). D: Over dominant model (Aa vs. AA + aa). E: AA vs. aa model. F: AA vs. Aa model.

Fourteen case-control studies involving 3650 participants reported a significant association between the *Apa*I polymorphism (rs7975232) in the allele contrast (OR = 1.18, 95 % CI = 1.06–1.30, *p* < 0.01, *I*^*2*^ = 55 %), the recessive model (OR = 1.27, 95 % CI = 1.05–1.53, *p* < 0.01, *I*^*2*^ = 63 %), the over dominant model (OR = 1.03, 95 % CI = 0.89–1.19, *p* < 0.01, *I*^*2*^ = 55 %), the AA vs. aa model (OR = 1.39, 95 % CI = 1.12–1.71, *p* < 0.01, *I*^*2*^ = 54 %), and the AA vs. Aa model (OR = 1.24, 95 % CI = 1.00–1.53, *p* < 0.01, *I*^*2*^ = 64 %), however, there was no significant association in the dominant model (OR = 1.21, 95 % CI = 1.05–1.39, *p* = 0.17, *I*^*2*^ = 27 %) (Fig. 2).

### 3.3 Polymorphism *Bsm*I (rs1544410)

**Fig. 3:**
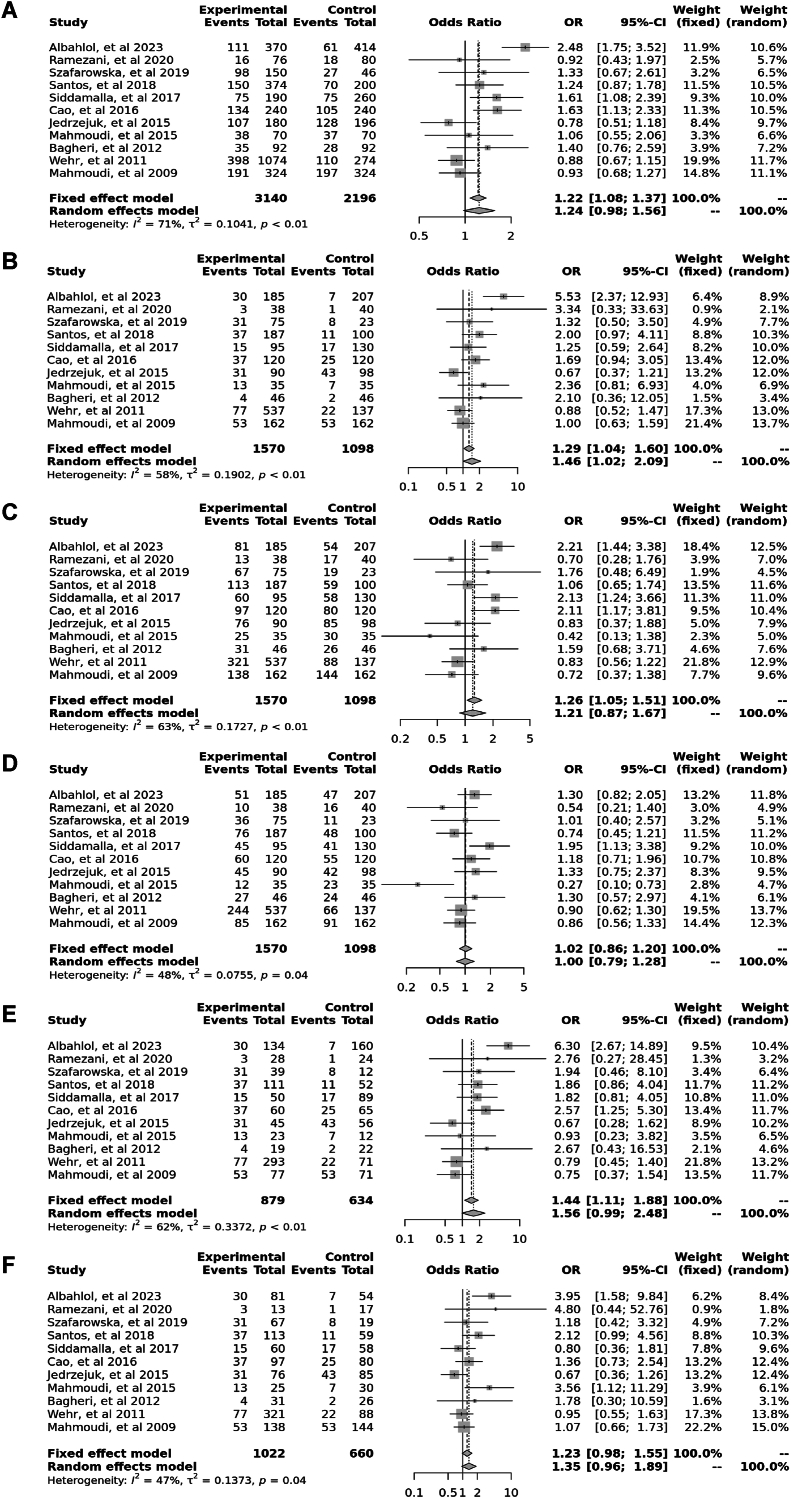
Forest plot illustrating the various genotypes and alleles in *Bsm*I (rs1544410) in PCOS women vs. control groups. A: Allele contrast (B vs. b). B: Recessive model (BB vs. Bb + bb). C: Dominant model (BB + Bb vs. bb). D: Over dominant model (Bb vs. BB + bb). E: BB vs. bb model. F: BB vs. Bb model.

Eleven case–control studies involving 2796 participants reported a significant association between the *Bsm*I polymorphism (rs1544410) in the allele contrast (OR = 1.22, 95 % CI = 1.08–1.37, *p* < 0.01, *I*^*2*^ = 71 %), the recessive model (OR = 1.29, 95 % CI = 1.04–1.60, *p* < 0.01, *I*^*2*^ = 58 %), the dominant model (OR = 1.26, 95 % CI = 1.05–1.51, *p* < 0.01, *I*^*2*^ = 63 %), the over dominant model (OR = 1.02, 95 % CI = 0.86–1.20, *p* = 0.04, *I*^*2*^ = 48 %), the BB vs. bb model (OR = 1.44, 95 % CI = 1.11–1.88, *p* < 0.01, *I*^*2*^ = 62 %), and the BB vs. Bb model (OR = 1.23, 95 % CI = 0.98–1.55, *p* < 0.04, *I*^*2*^ = 47 %) (Fig. 3).

### 3.4 Polymorphism Cdx2 (rs11568820)

**Fig. 4:**
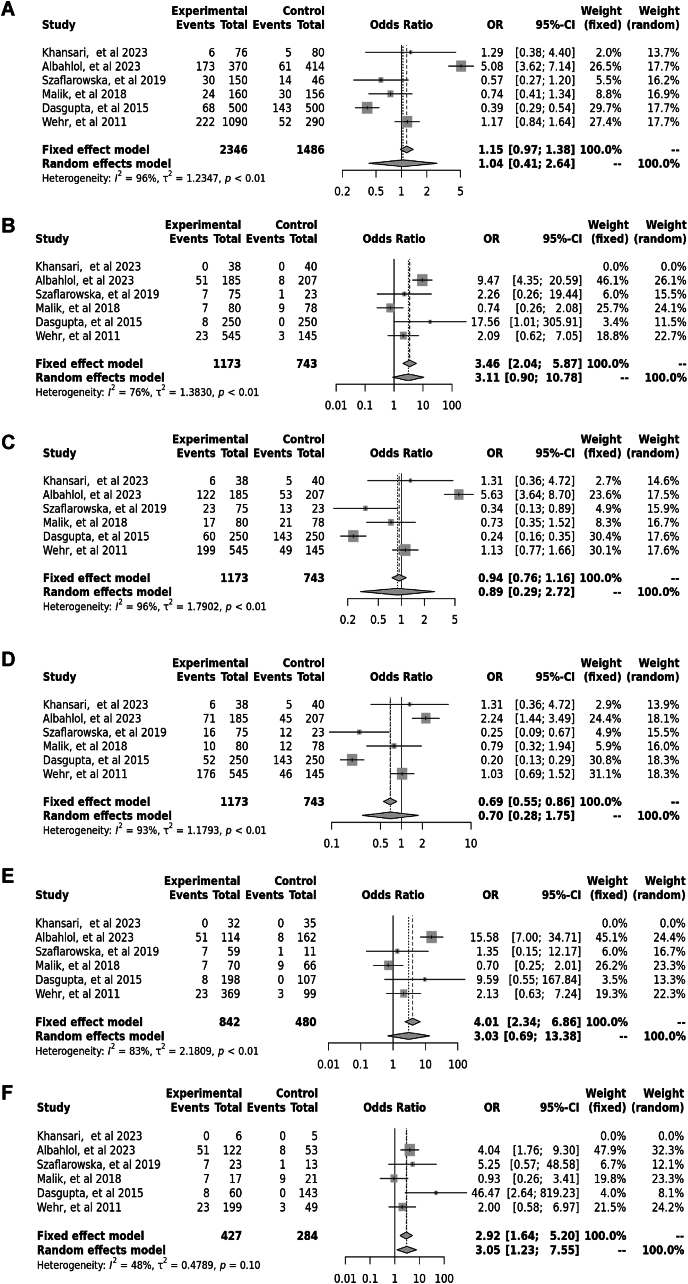
Forest plot illustrating the various genotypes and alleles in Cdx2 (rs11568820) in PCOS women vs. control groups. A: Allele contrast (C vs. c). B: Recessive model (CC vs. *Cc* + cc). C: Dominant model (CC + *Cc* vs. cc). D: Over dominant model (*Cc* vs. CC + cc). E: CC vs. cc model. F: CC vs. *Cc* model.

Six case–control studies involving 2041 participants reported a significant association between the Cdx2 polymorphism (rs11568820) in the allele contrast (OR = 1.15, 95 % CI = 0.97–1.38, *p* < 0.01, *I*^*2*^ = 96 %), the recessive model (OR = 3.46, 95 % CI = 2.04–5.87, *p* < 0.01, *I*^*2*^ = 76 %), the dominant model (OR = 0.94, 95 % CI = 0.76–1.16, *p* < 0.01, *I*^*2*^ = 96 %), the over dominant model (OR = 0.69, 95 % CI = 0.55–0.86, *p* < 0.01, *I*^*2*^ = 93 %), the CC vs. cc model (OR = 4.01, 95 % CI = 2.34–6.86, *p* < 0.01, *I*^*2*^ = 83 %), and the CC vs. *Cc* model (OR = 2.92, 95 % CI = 1.67–5.20, *p* < 0.01, *I*^*2*^ = 48 %) (Fig. 4).

### 3.5 Polymorphism *Fok*I (rs22228570)

**Fig. 5:**
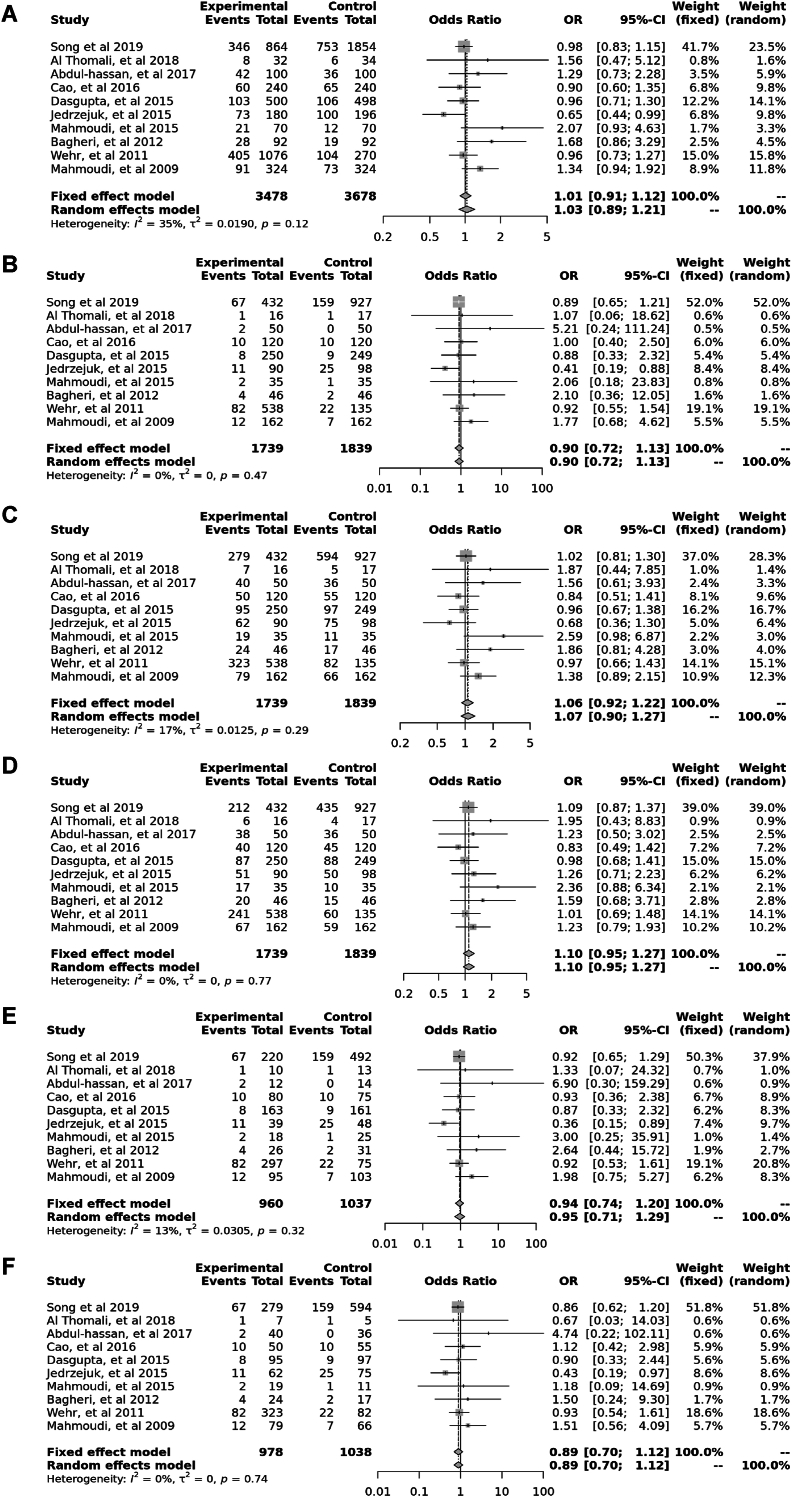
Forest plot illustrating the various genotypes and alleles in *Fok*I (rs2228570) in PCOS women vs. control groups. A: Allele contrast (F vs. f). B: Recessive model (FF vs. Ff + ff). C: Dominant model (FF + Ff vs. ff). D: Over dominant model (Ff vs. FF + ff). E: FF vs. ff model. F: FF vs. Ff model.

Ten case–control studies involving 3705 participants reported no significant association between *Fok*I (rs22228570) polymorphism in allele contrast (OR = 1.01, 95 % CI = 0.91–1.112, *p* = 0.12, *I*^2^ = 35 %), the recessive model (OR = 0.90, 95 % CI = 0.72–1.13, *p* = 0.47, *I*^2^ = 0 %), the dominant model (OR = 1.06, 95 % CI = 0.92–1.22, *p* = 0.29, *I*^2^ = 17 %), the over-dominant model (OR = 1.01, 95 % CI = 0.95–1.27, *p* = 0.77, *I*^2^ = 0 %), the FF vs. ff model (OR = 0.94, 95 % CI = 0.74–1.20, *p* = 0.32, *I*^2^ = 13 %), and the FF vs. Ff model (OR = 0.89, 95 % CI = 0.70–1.12, *p* = 0.74, *I*^2^ = 0 %) (Fig. 5).

### 3.6 Polymorphism *Taq*I (rs731236)

**Fig. 6:**
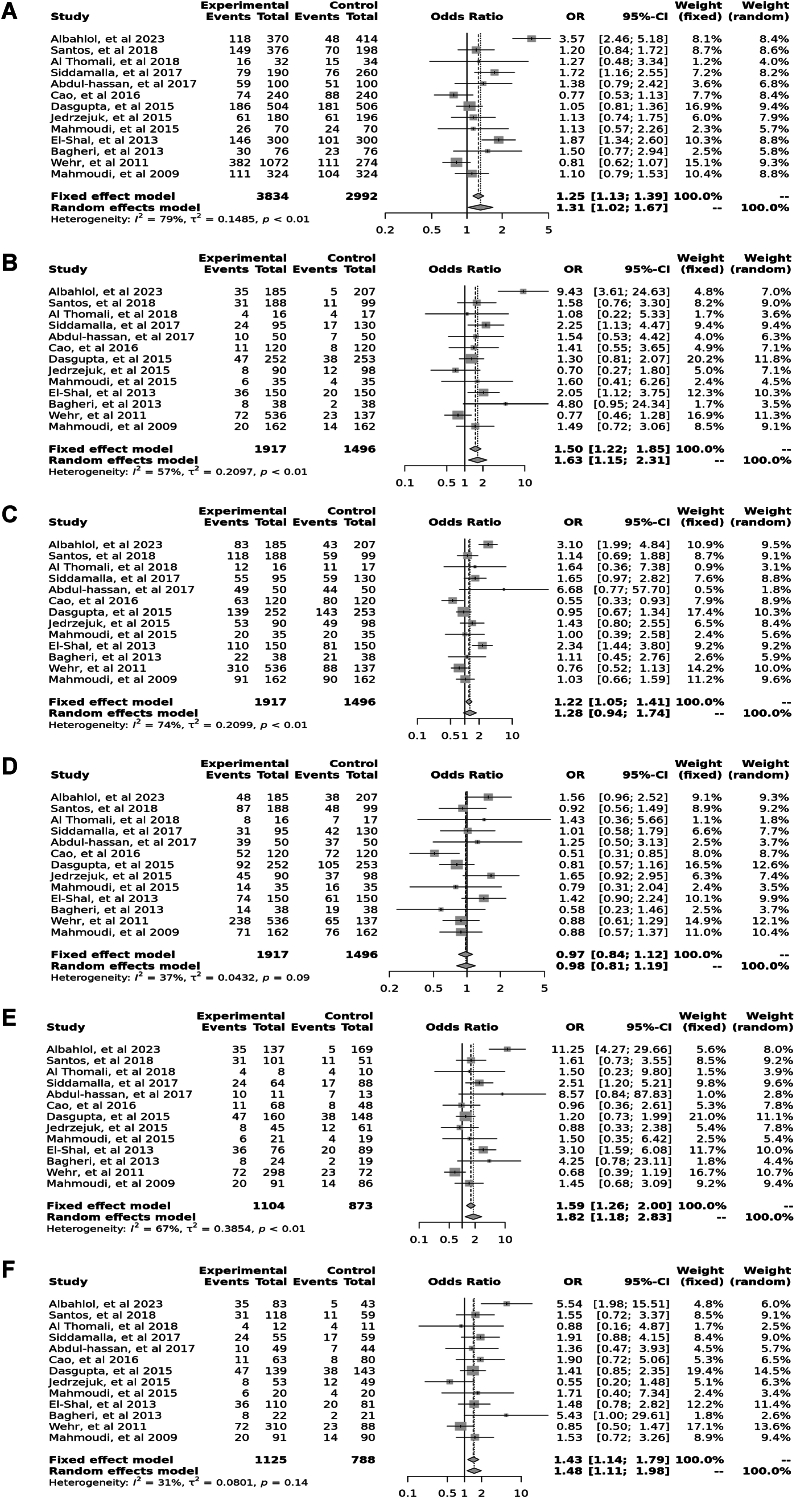
Forest plot illustrating the various genotypes and alleles in *Taq*I (rs731236) in PCOS women vs. control groups. A: Allele contrast (T vs. t). B: Recessive model (TT vs. Tt + tt). C: Dominant model (TT + Tt vs. tt). D: Over dominant model (Tt vs. TT + tt). E: TT vs. tt model. F: TT vs. Tt model.

Thirteen case–control studies involving 3538 participants reported a significant association between the Taq polymorphism (rs731236) in allele contrast (OR = 1.25, 95 % CI = 1.13–1.39, *p* < 0.01, *I*^2^ = 79 %), the recessive model (OR = 1.50, 95 % CI = 1.22–1.85, *p* < 0.01, *I*^2^ = 57 %), the dominant model (OR = 1.22, 95 % CI = 1.05–1.41, *p* < 0.01, *I*^2^ = 74 %), and the TT vs. tt model (OR = 1.59, 95 % CI = 1.26–2.00, *p* < 0.01, *I*^2^ = 67 %), however there was no significant association in the over dominant model (OR = 0.97, 95 % CI = 0.84–1.12, *p* = 0.09, *I*^2^ = 37 %), and the TT vs. Tt model (OR = 1.43, 95 % CI = 1.14–1.79, *p* = 0.14) (Fig. 6).

### 3.7 Heterogeneity, publication bias, and sensitivity analysis

Specific genetic models, particularly the *Bsm*I SNP, showed heterogeneity. Egger's tests indicated an absence of publication bias. The outcomes of the sensitivity analysis were illustrated in funnel plots and sensitivity plots (Fig. 7, Fig. 8, Fig. 9, Fig. 10, Fig. 11, Fig. 12, Fig. 13, Fig. 14, Fig. 15, Fig. 16).

**Fig. 7:**
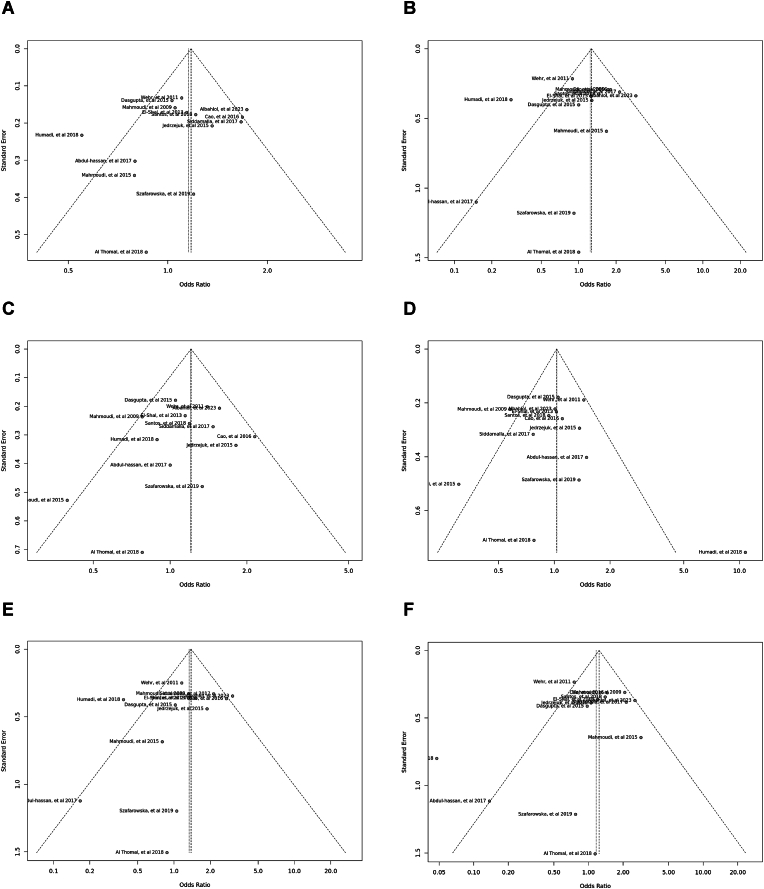
Funnel plot illustrating the various genotypes and alleles in *Apa*I (rs7975232) in PCOS women vs. control groups. A: Allele contrast (A vs. a). B: Recessive model (AA vs. Aa + aa). C: Dominant model (AA + Aa vs. aa). D: Over dominant model (Aa vs. AA + aa). E: AA vs. aa model. F: AA vs. Aa model.

**Fig. 8:**
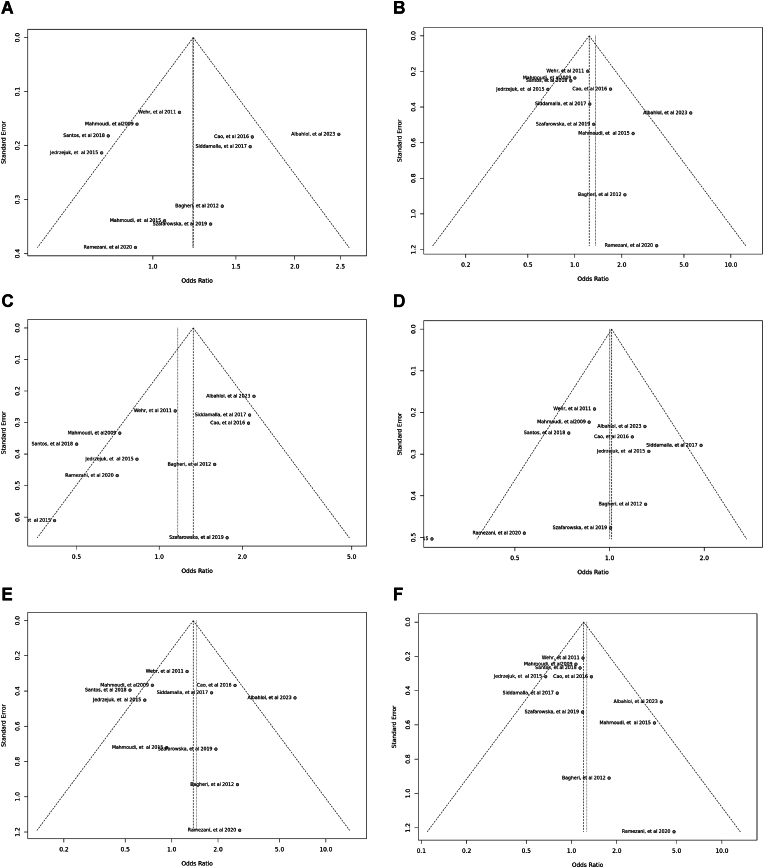
Funnel plot illustrating the various genotypes and alleles in *Bsm*I (rs1544410) in PCOS women vs. control groups. A: Allele contrast (B vs. b). B: Recessive model (BB vs. Bb + bb). C: Dominant model (BB + Bb vs. bb). D: Over dominant model (Bb vs. BB + bb). E: BB vs. bb model. F: BB vs. Bb model.

**Fig. 9:**
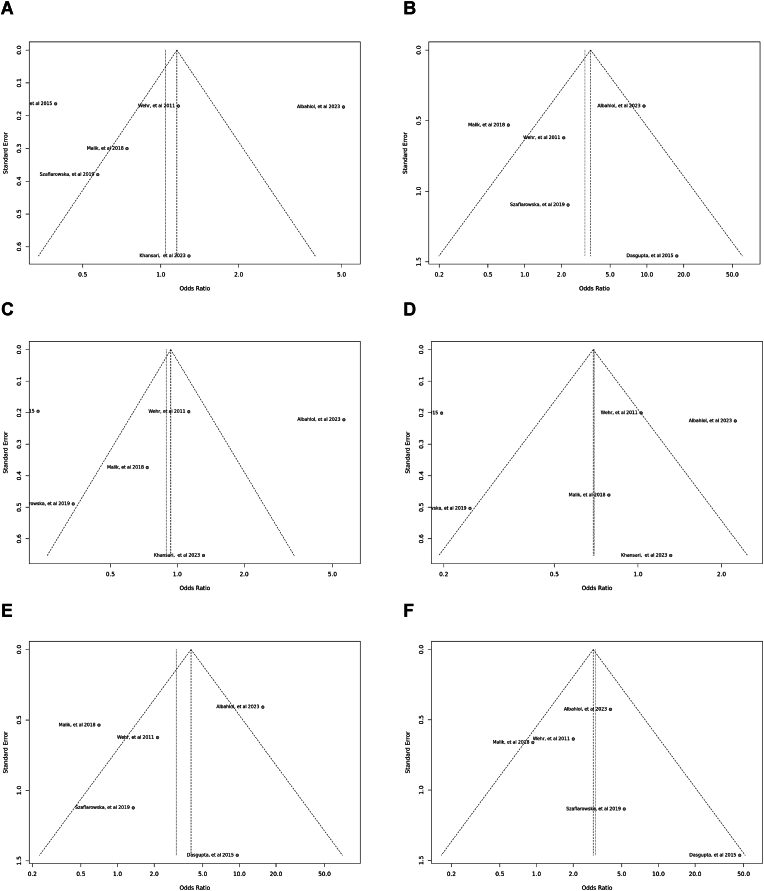
Funnel plot illustrating the various genotypes and alleles in Cdx2 (rs11568820) in PCOS women vs. control groups. A: Allele contrast (C vs. c). B: Recessive model (CC vs. *Cc* + cc). C: Dominant model (CC + *Cc* vs. cc). D: Over dominant model (*Cc* vs. CC + cc). E: CC vs. cc model. F: CC vs. *Cc* model.

**Fig. 10:**
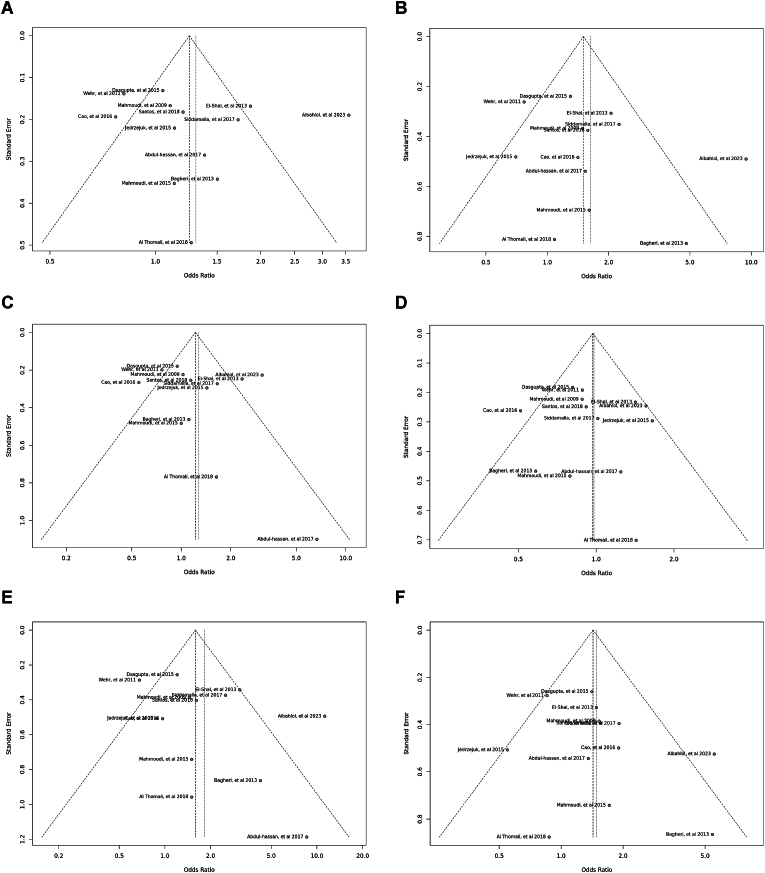
Funnel plot illustrating the various genotypes and alleles in *Fok*I (rs2228570) in PCOS women vs. control groups. A: Allele contrast (F vs. f). B: Recessive model (FF vs. Ff + ff). C: Dominant model (FF + Ff vs. ff). D: Over dominant model (Ff vs. FF + ff). E: FF vs. ff model. F: FF vs. Ff model.

**Fig. 11:**
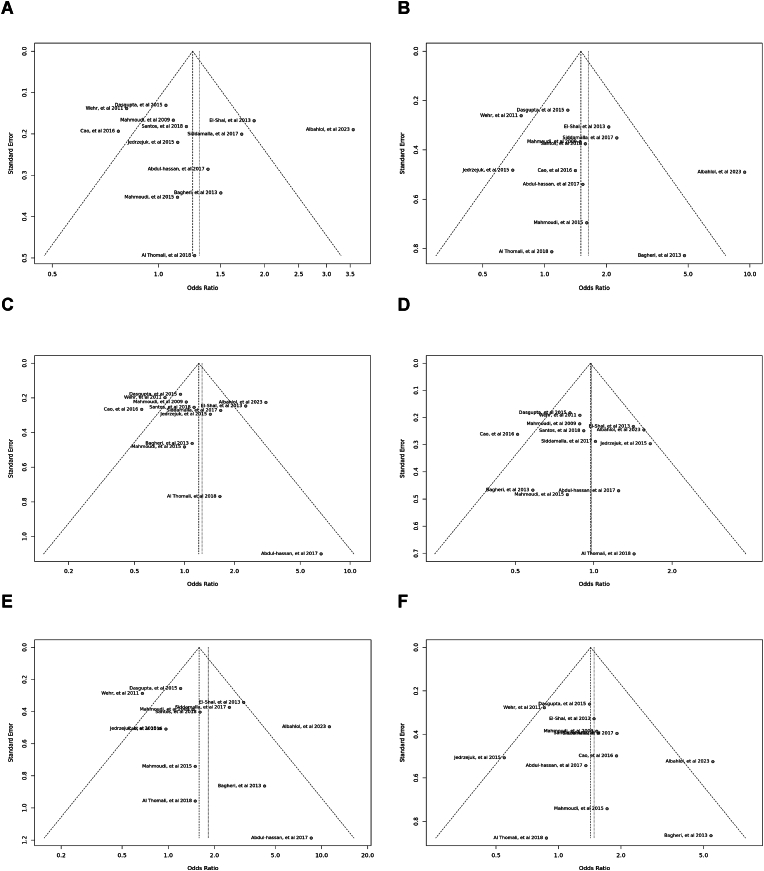
Funnel plot illustrating the various genotypes and alleles in *Taq*I (rs731236) in PCOS women vs. control groups. A: Allele contrast (T vs. t). B: Recessive model (TT vs. Tt + tt). C: Dominant model (TT + Tt vs. tt). D: Over dominant model (Tt vs. TT + tt). E: TT vs. tt model. F: TT vs. Tt model.

**Fig. 12:**
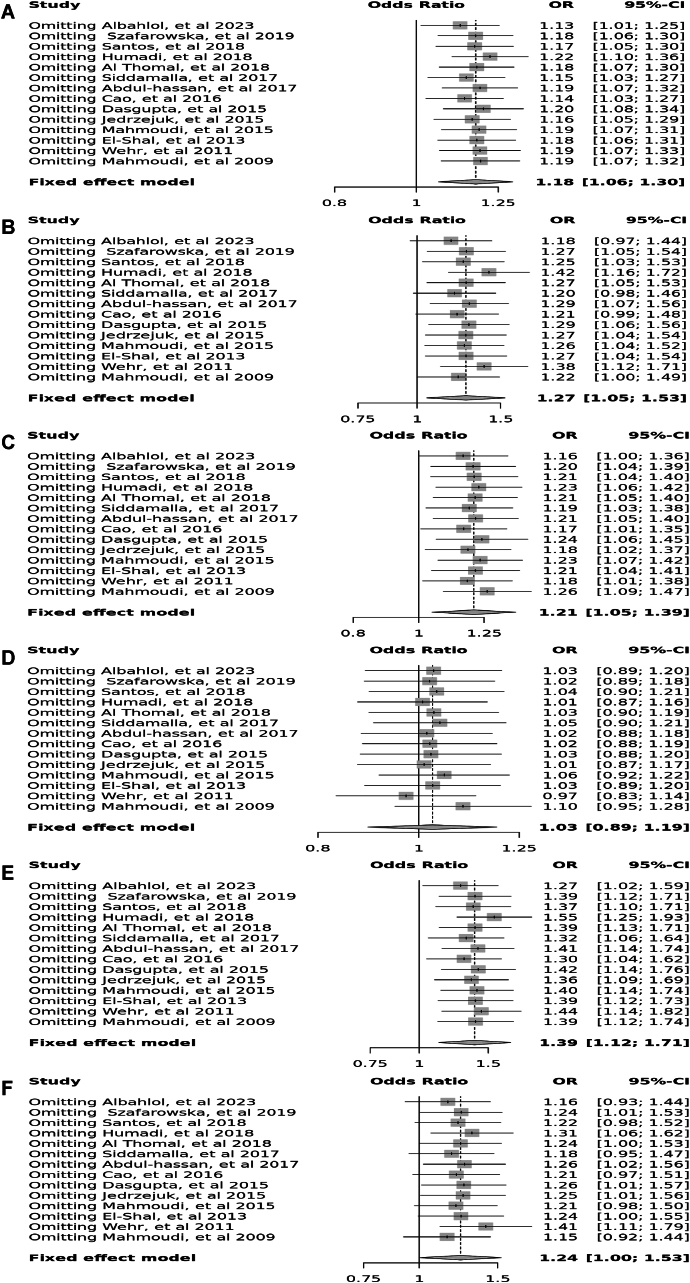
Sensitivity plot illustrating the various genotypes and alleles in *Apa*I (rs7975232) in PCOS women vs. control groups. A: Allele contrast (A vs. a). B: Recessive model (AA vs. Aa + aa). C: Dominant model (AA + Aa vs. aa). D: Over dominant model (Aa vs. AA + aa). E: AA vs. aa model. F: AA vs. Aa model.

**Fig. 13:**
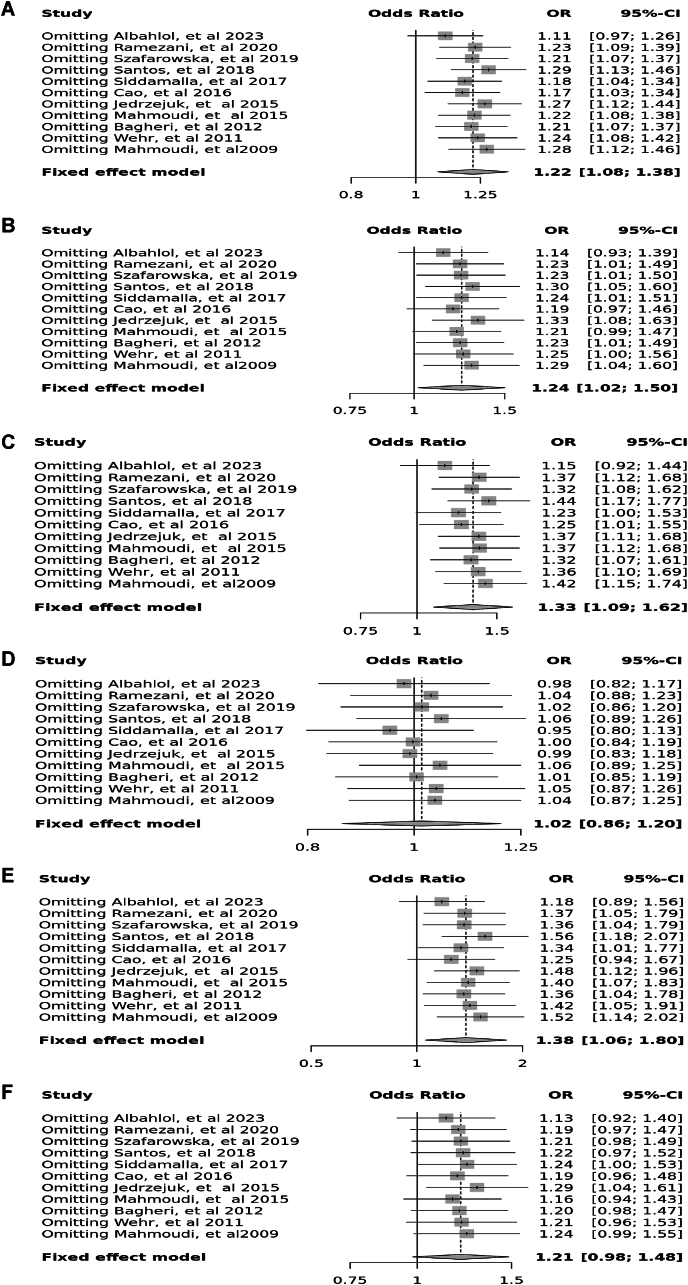
Sensitivity plot illustrating the various genotypes and alleles in *Bsm*I (rs1544410) in PCOS women vs. control groups. A: Allele contrast (B vs. b). B: Recessive model (BB vs. Bb + bb). C: Dominant model (BB + Bb vs. bb). D: Over dominant model (Bb vs. BB + bb). E: BB vs. bb model. F: BB vs. Bb model.

**Fig. 14:**
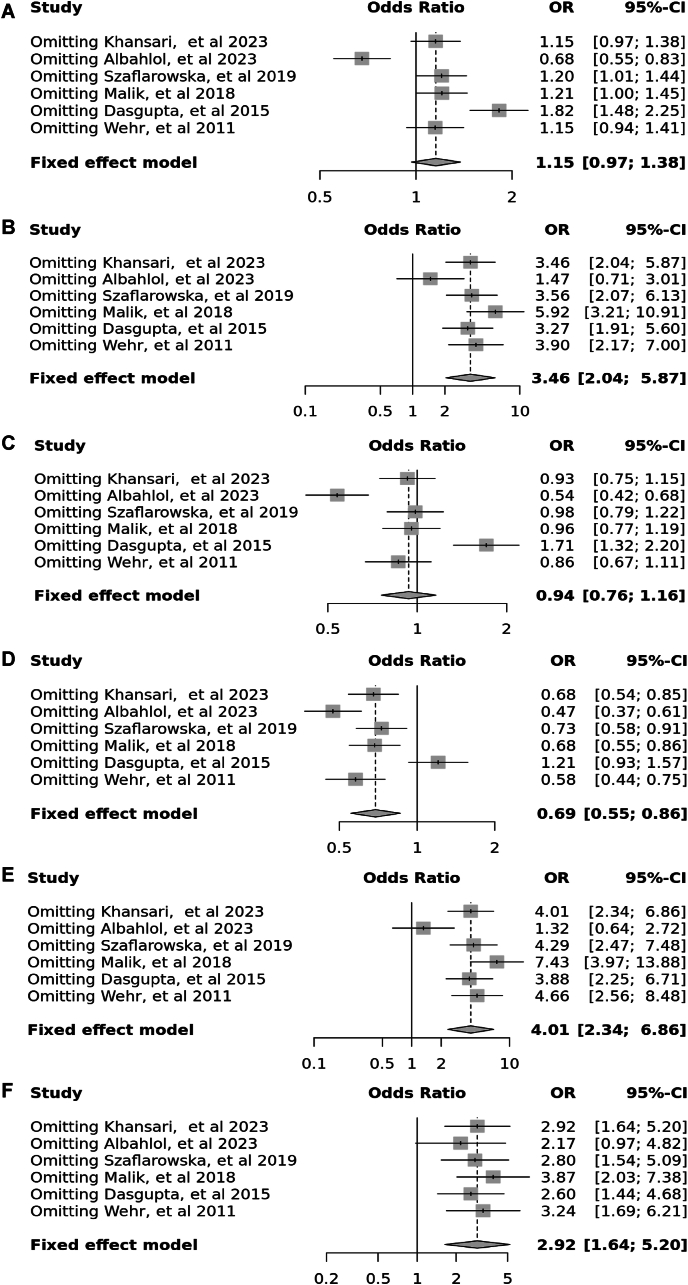
Sensitivity plot illustrating the various genotypes and alleles in Cdx2 (rs11568820) in PCOS women vs. control groups. A: Allele contrast (C vs. c). B: Recessive model (CC vs. *Cc* + cc). C: Dominant model (CC + *Cc* vs. cc). D: Over dominant model (*Cc* vs. CC + cc). E: CC vs. cc model. F: CC vs. *Cc* model.

**Fig. 15:**
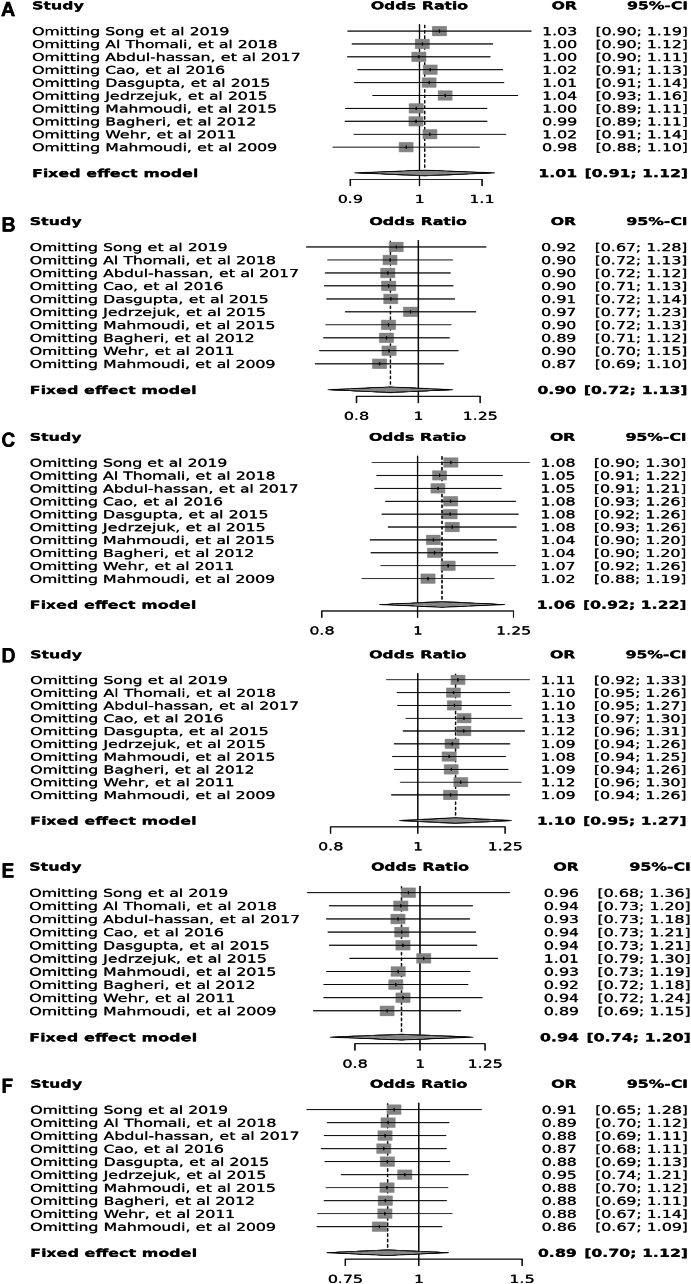
Sensitivity plot illustrating the various genotypes and alleles in *Fok*I (rs2228570) in PCOS women vs. control groups. A: Allele contrast (F vs. f). B: Recessive model (FF vs. Ff + ff). C: Dominant model (FF + Ff vs. ff). D: Over dominant model (Ff vs. FF + ff). E: FF vs. ff model. F: FF vs. Ff model.

**Fig. 16:**
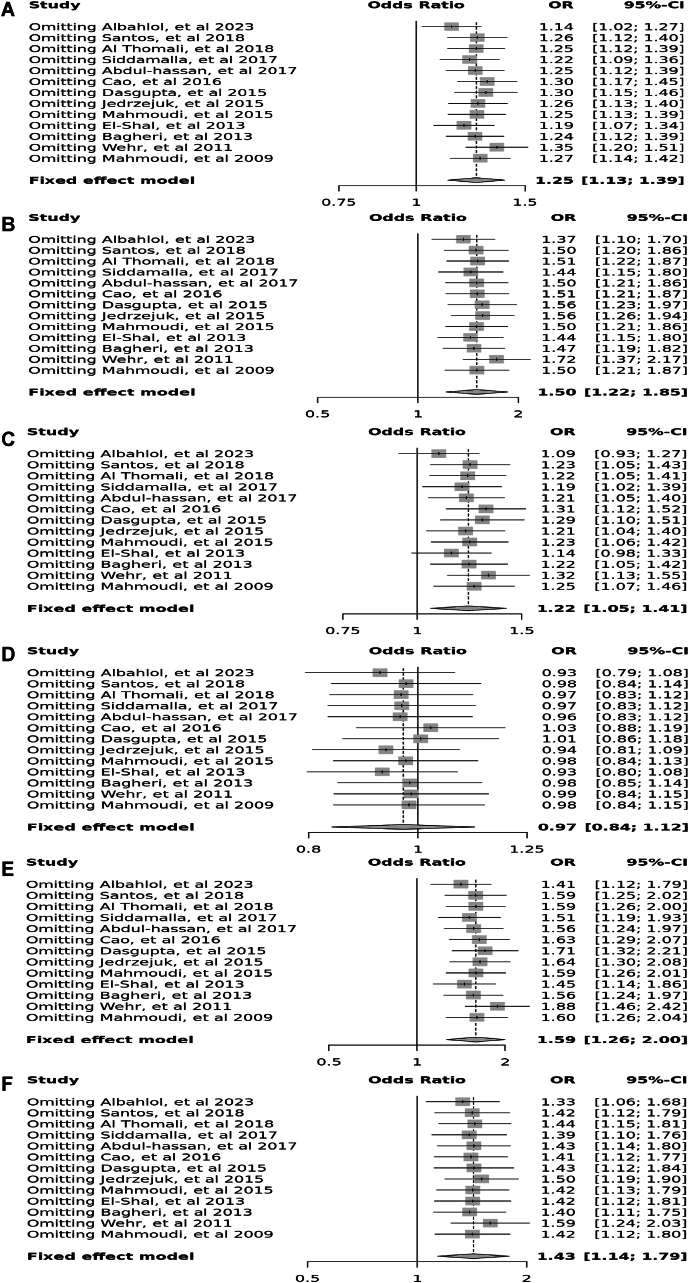
Sensitivity plot illustrating the various genotypes and alleles in *Taq*I (rs731236) in PCOS women vs. control groups. A: Allele contrast (T vs. t). B: Recessive model (TT vs. Tt + tt). C: Dominant model (TT + Tt vs. tt). D: Over dominant model (Tt vs. TT + tt). E: TT vs. tt model. F: TT vs. Tt model.

### 3.8 Subgroup analysis based on the ethnicity differences

A subgroup analysis was conducted to investigate the potential influence of patient BMI and ethnicity on the association between VDR gene polymorphisms and PCOS risk. The analysis of the *Apa*I (rs7975232) gene polymorphism in overweight and obese individuals revealed a significant association in the allele contrast (OR = 1.20, 95 % CI = 1.03–1.39, *p* = 0.018), the recessive model (OR = 1.57, 95 % CI = 1.19–2.07, *p* = 0.001), the dominant model (OR = 1.09, 95 % CI = 0.87–1.36, *p* = 0.414), the pairw1 (AA vs. aa) model (OR = 1.52, 95 % CI = 1.12–2.07, *p* = 0.006), and the pairw2 (AA vs. Aa) model (OR = 1.59, 95 % CI = 1.17–2.15, *p* = 0.002). Caucasian individuals exhibited significant associations in the allele contrast (OR = 1.26, 95 % CI = 1.09–1.46, *p* = 0.001), recessive model (OR = 1.28, 95 % CI = 0.98–1.66, *p* = 0.062), dominant model (OR = 1.37, 95 % CI = 1.12–1.68, *p* = 0.001), and pairw1 (AA vs. aa) model (OR = 1.56, 95 % CI = 1.16–2.09, *p* = 0.002) for the *Apa*I (rs7975232) gene polymorphism. Analysis of the *Bsm*I (rs1544410) polymorphism showed significant associations in Asians for allele contrast (OR = 1.24, 95 % CI = 1.04–1.49, *p* = 0.015), the recessive model (OR = 1.34, 95 % CI = 0.98–1.81, *p* = 0.060), the dominant model (OR = 1.34, 95 % CI = 1.00–1.80, *p* = 0.043), and the pairw1 (AA vs. aa) model (OR = 1.50, 95 % CI = 1.01–2.24, *p* = 0.041). The *Taq*I (rs731236) polymorphism analysis indicated a significant association in Caucasians for the allele contrast (OR = 1.39, 95 % CI = 1.19–1.61, *p* = 2.12E-05), the recessive model (OR = 1.43, 95 % CI = 1.05–1.95, *p* = 0.020), the dominant model (OR = 1.42, 95 % CI = 1.03–1.95, *p* = 0.030), and the pairw2 (AA vs. Aa) model (OR = 1.25, 95 % CI = 0.90–1.74, *p* = 0.165).

## 4 Discussion

The mechanism of action of vitamin D is mediated by the VDR, a ligand-dependent transcription factor that is classified within the steroid/thyroid hormone receptor superfamily [7]. Vitamin D controls the female reproductive system by supporting the production of steroids and related hormones, including AMH, follicle-stimulating hormone, progesterone in GCs, and glucose hemostasis in pancreatic β-cells [8]. The VDR gene is found on chromosome 12q13.11 and is comprised of 14 exons that are responsible for encoding a protein that is 427 amino acids in length [9]. A total of five SNPs has been found to be present in the VDR gene. These SNPs include *Apa*I (rs7975232) situated in intron 8, *Bsm*I (rs1544410) located in intron 8, Cdx2 (rs11568820) located in exon 1, *Fok*I (rs10735810) located in exon 2, and *Taq*I (rs731236) placed in exon 9 [9]. It is essential to get an understanding of the relationships between VDR gene polymorphisms and the risk of developing PCOS and infertility in order to improve the diagnosis, treatment, and management of this disorder. There is a correlation between VDR gene polymorphisms and the risk of PCOS and infertility, according to the data that has been criticized [10]. On the other hand, the association between the VDR gene polymorphisms and PCOS remained unclear. *Apa*I, *Bsm*I, and *Fok*I are only a few of the VDR gene polymorphisms that have been identified as having a role in the development of PCOS by previous meta-analyses [11].

The present systematic review and meta-analysis focused on five important VDR gene polymorphisms, including *Apa*I (rs7975232), *Bsm*I (rs1544410), Cdx2 (rs11568820), *Fok*I (rs22228570), and *Taq*I (rs731236), as well as the risk for the development of PCOS. The total number of participants in the study was 5618. An updated meta-analysis that incorporated more population studies might address research gaps in VDR gene polymorphisms as well as the risk for PCOS and infertility. VDR gene polymorphisms were shown to be significantly related to an elevated risk of PCOS and infertility in females, according to the findings of our study.

Collectively, our results suggested a significant association between the existence of *Apa*I (rs7975232) polymorphism and an increased risk of developing PCOS and infertility. Supporting our findings, prior meta-analyses have shown a significant correlation between *Apa*I and heightened susceptibility to PCOS [11,33]. In Shi et al.'s meta-analysis, 2825 cases and controls were examined for a substantial association between the existence of *Apa*I (rs7975232) polymorphism and susceptibility to PCOS (OR = 1.19, 95 % CI = 1.06–1.34, *p* = 0.004) [11]. According to a previous meta-analysis, 1027 case and control participants were included in the Shahmoradi et al. meta-analysis to investigate whether there was a significant association between the presence of the *Apa*I (rs7975232) polymorphism and PCOS susceptibility (OR = 1.466, 95 % CI = 1.09–1.97, *p* = 0.01) [33]. Our meta-analysis was conducted on a relatively larger sample size consisting of 3650 cases and controls with a significant association between the *Apa*I polymorphism (rs7975232) in the allele contrast (OR = 1.18, 95 % CI = 1.06–1.30, *p* < 0.01), the recessive model (OR = 1.27, 95 % CI = 1.05–1.53, p < 0.01), the over dominant model (OR = 1.03, 95 % CI = 0.89–1.19, *p* < 0.01), and the AA vs. aa model (OR = 1.24, 95 % CI = 1.00–1.53, *p* < 0.01); however, there was no significant association in the dominant model (OR = 1.21, 95 % CI = 1.05–1.39, *p* = 0.17).

In addition, the findings of our study indicated a strong association between the presence of the *Bsm*I (rs1544410) polymorphism and a higher susceptibility to developing PCOS and infertility. Corroborating our results, previous studies have shown a substantial association between *Bsm*I and increased vulnerability to PCOS [11,34]. In Shi et al.'s meta-analysis, 2099 cases and controls were examined for a substantial association between the existence of the *Bsm*I (rs1544410) polymorphism and susceptibility to PCOS (OR = 1.27, 95 % CI = 1.06–1.53, *p* = 0.011) [11]. In accordance with a previous meta-analysis, 1813 case and control participants were included in the Niu et al. meta-analysis to investigate whether there was a significant association between the presence of the *Bsm*I (rs1544410) polymorphism and PCOS susceptibility (OR = 1.62, 95%CI = 1.24–2.11, *p* < 0.01) [34]. The present meta-analysis was performed on a comparatively larger sample set, including 2796 participants, and reported a significant association between the *Bsm*I polymorphism (rs1544410), (OR = 1.22, 95 % CI = 1.08–1.37, *p* < 0.01, *I*^2^ = 71 %), the recessive model (OR = 1.29, 95 % CI = 1.04–1.60, *p* < 0.01, *I*^2^ = 58 %), the dominant model (OR = 1.26, 95 % CI = 1.05–1.51, p < 0.01, *I*^2^ = 63 %), the over dominant model (OR = 1.02, 95 % CI = 0.86–1.20, *p* = 0.04, *I*^2^ = 48 %), the BB vs. bb model (OR = 1.44, 95 % CI = 1.11–1.88, *p* < 0.01, *I*^2^ = 62 %), and the BB vs. Bb model (OR = 1.23, 95 % CI = 0.98–1.55, *p* < 0.04, *I*^2^ = 47 %).

Furthermore, the results of our investigation revealed a significant correlation between the existence of the Cdx2 (rs11568820) polymorphism and an increased vulnerability to the development of PCOS and infertility. Consistent with our findings, prior research has shown a significant correlation between Cdx2 and heightened susceptibility to PCOS in the Egyptian population [9]. However, a case-control study in the Indian population revealed a protective relationship between the presence of the Cdx2 (rs11568820) polymorphism and PCOS [24]. Other studies on Pakistani women showed a non-significant association between Cdx2 (rs11568820) polymorphism and PCOS [21]. A larger sample size of 2041 individuals was used for our meta-analysis, which revealed a substantial association between the Cdx2 polymorphism (rs11568820) in the allele contrast (OR = 1.15, 95 % CI = 0.97–1.38, *p* < 0.01), the recessive model (OR = 3.46, 95 % CI = 2.04–5.87, *p* < 0.01), the dominant model (OR = 0.94, 95 % CI = 0.76–1.16, *p* < 0.01), the over dominant model (OR = 0.69, 95 % CI = 0.55–0.86, *p* < 0.01), the CC vs. cc model (OR = 0.69, 95 % CI = 0.55–0.86, *p* < 0.01), and the CC vs. *Cc* model (OR = 4.01, 95 % CI = 2.34–6.86, *p* < 0.01).

The results of the Cdx2 polymorphism show considerable discrepancies. Variations across groups may arise from disparities in genetic backgrounds, including allele frequencies, haplotype configurations, and linkage disequilibrium patterns. Furthermore, dietary habits, BMI, and other environmental variables may alter VDR activity and interact with Cdx2 polymorphism to influence PCOS risk. These factors may include vitamin D status, which is regulated by food consumption, supplementation, and sunshine exposure. These relationships underscore the intricate nature of PCOS as a complex condition. To rectify these discrepancies, further studies are needed for forthcoming investigations that include varied populations and extensive data on confounding variables to clarify the function of Cdx2 polymorphism in the etiology of PCOS.

Moreover, our study's findings demonstrated no significant association between the *Fok*I (rs22228570) polymorphism and an increased susceptibility to PCOS or infertility. Consistent with our results, a previous meta-analysis by Shi et al. that included 2086 case and control participants reported no link between the *Fok*I (rs22228570) polymorphism and risk for PCOS [10]. Similarly, case-control studies conducted in PCOS vs. non-PCOS women in Australia [31], Poland [25], India [24], and Korea [17] reported comparable results. In our meta-analysis, a total of 3705 participants were included. No significant associations were observed for the *Fok*I (rs22228570) polymorphism across various genetic models: allele contrast (OR = 1.01, 95 % CI = 0.91–1.112, *p* = 0.12), recessive model (OR = 0.90, 95 % CI = 0.72–1.13, *p* = 0.47), dominant model (OR = 1.06, 95 % CI = 0.92–1.22, *p* = 0.29), over-dominant model (OR = 1.01, 95 % CI = 0.95–1.27, *p* = 0.77), FF vs. ff model (OR = 0.94, 95 % CI = 0.74–1.20, *p* = 0.32), FF vs. Ff model (OR = 0.89, 95 % CI = 0.70–1.12, *p* = 0.74).

Meanwhile, the results of our research revealed a substantial association between the *Taq*I (rs731236) polymorphism and a heightened vulnerability to PCOS or infertility. In contrast with our meta-analysis, previous meta-analyses included 3036 case and control participants and reported no associations between *Taq*I (rs731236) polymorphism and PCOS in women [11]. Thirteen case–control studies involving 3538 participants reported a significant association between the Taq polymorphism (rs731236) in the allele contrast (OR = 1.25, 95 % CI = 1.13–1.39, *p* < 0.01), the recessive model (OR = 1.50, 95 % CI = 1.22–1.85, *p* < 0.01), the dominant model (OR = 1.22, 95 % CI = 1.05–1.41, *p* < 0.01) and the TT vs. tt model (OR = 1.59, 95 % CI = 1.26–2.00, *P* < 0.01), however there was no significant association in the over dominant model (OR = 0.97, 95 % CI = 0.84–1.12, *P* = 0.09) and the TT vs. Tt model (OR = 1.43, 95 % CI = 1.14–1.79, *p* = 0.14).

A subgroup analysis was also undertaken to investigate the possible significance of patient BMI and ethnicity on the association between VDR gene polymorphisms and the risk of PCOS. Significant associations were found for specific polymorphisms within certain subgroups, suggesting that both BMI and ethnicity may play a role in modifying these associations. For instance, the analysis of the *Apa*I (rs7975232) polymorphism in overweight and obese individuals revealed a significant association in the allele contrast model (OR = 1.20, 95 % CI = 1.03–1.39, p = 0.018). Similarly, analysis of the *Bsm*I (rs1544410) polymorphism showed significant associations in Asians for the allele contrast model (OR = 1.24, 95 % CI = 1.04–1.49, p = 0.015). This finding could be explained by genetic differences between ethnic groupings. Furthermore, due to the procedure of natural selection, functional variations in various groups may differ [35].

While Egger's tests generally indicated an absence of publication bias, some asymmetry was observed in the funnel plots for specific genetic models, suggesting potential publication bias. For instance, in the *Bsm*I (rs1544410) polymorphism, there is noticeable asymmetry in the allele contrast (B vs. b) and the recessive model (BB vs. Bb + bb). This suggests that studies with smaller sample sizes and less significant results might be underrepresented. However, it is crucial to recognize that funnel plot asymmetry does not definitively prove publication bias. Other factors, such as heterogeneity between studies or true differences in effect size across studies with varying sample sizes, could also contribute to the observed asymmetry. Further investigation and careful interpretation are necessary to determine the extent of publication bias in these cases.

The present systematic review and meta-analysis give important information regarding the association of VDR gene polymorphisms with PCOS. However, confounding factors, such as those not discussed broadly in the involved studies, may have an effect on the results. Important among the modulators of VDR activity that may interact with VDR gene polymorphisms to affect PCOS susceptibility include circulating vitamin D level, dietary habits, sun exposure, and body mass index. As an example, vitamin D status might impact immune and endocrine functions controlled by VDR [36]. Vitamin D executes its critical function in immune and endocrine modalities through its active metabolite, 1,25-dihydroxyvitamin D3 (1,25(OH)₂D₃), via the vitamin D receptor VDR [37]. This modulation has a vital role in the health of the immune system and a wide implication in diseases. VDR is highly expressed on numerous significant immune cells of the immune system, which include B and T lymphocytes, monocytes, macrophages, and dendritic cells [[37], [38], [39]]. These cells can, amongst other functions, locally convert vitamin D into its active form, which may be of great importance for the modulation of immune responses [40]. Vitamin D strengthens innate immunity by enhancing the production of antimicrobial peptides and supports adaptive immunity by modulating T cell responses. Thus, it hinders inflammatory TH cells and enhances Treg cells, being important for the prevention of autoimmunity [37,39]. Anti-inflammatory effects of vitamin D signaling via VDR include a decrease in the secretion of inflammatory cytokines and induction of immune tolerance [37,40]. Being a nuclear receptor, VDR controls the expression of more than 900 genes, covering a wide range of physiological processes, including immune and endocrine functions [36,41]. Besides its well-documented role in the regulation of calcium and phosphate homeostasis, vitamin D exerts an impact on other endocrine systems, thus contributing to the maintenance of health and prevention of diseases [38]. Vitamin D, via interaction with VDR, may play an important role in immune and endocrine functions [36,39]. It keeps the balance of immune homeostasis through a potent enhancement of antimicrobial defense and modulation of inflammatory responses and is involved in endocrine health [37,41]. A fair level of the vitamin is needed to prevent many diseases or for normal health conditions [41].

Several studies have found the influence of VDR polymorphisms on insulin resistance in the background of PCOS. Thus, for instance, the Cdx2 genotype is associated with better insulin sensitivity; 'AA' genotype carriers are characterized by decreased levels of fasting insulin and better indices of insulin sensitivity in the group of women with PCOS [42,43]. There is also a meaningful influence of *Taq*I polymorphism on insulin resistance in PCOS, particularly detected in some ethnic groups [43,44]. Insulin resistance in the background of PCOS partially depends on increased serine phosphorylation of an insulin receptor that influences insulin signaling pathways. Such a defect is probably dependent on the modulation of VDR polymorphism that may influence insulin receptor signaling pathways and contribute to the metabolic phenotype of PCOS [45]. Less evident is the direct link between VDR polymorphisms and disturbances in anti-Müllerian hormone regulation in PCOS. However, because VDR participates in more general regulation of steroidogenesis, the indirect impact should be considered. Thus, VDR polymorphisms have been associated with altered levels of testosterone, which might influence AMH production and ovarian function [42]. The effect of VDR polymorphisms on PCOS varies among ethnic groups, where some variants were found to be strongly associated in Asians and Caucasians. This variability likely hints at much-needed research in diverse populations to finally clarify the genetic mystery behind PCOS [43].

Although the present meta-analysis throws some light on the correlation between VDR gene polymorphisms and PCOS, several limitations must be pointed out. Most of the studies included in this meta-analysis were region-specific, which limits the generalizability of our findings. Most of the studies were conducted in countries like Egypt, Poland, Brazil, Iraq, Saudi Arabia, India, China, and Australia. More global representation will give an edge in terms of wider applicability. Moreover, confounding variables such as circulating levels of vitamin D, dietary habits, sun exposure, and body mass index, which are significant modifiers of VDR activity, have not been properly addressed in the subjects studied. We recommend that future studies consider these factors to provide a better mechanistic understanding of VDR polymorphisms in PCOS. Current results are from relatively small sample sizes, thus emphasizing the need for further, larger, and more diverse studies. We strongly recommend GWAS for the identification of other genetic markers and also to study the interplay between genetic and environmental factors involved in the manifestation of PCOS.

## 5 Conclusion

This systematic review and meta-analysis provide essential information on the association of VDR gene polymorphisms with PCOS in women. It was particularly noted that women with *Apa*I, *Bsm*I, Cdx2, and *Taq*I polymorphisms of the VDR gene may be more susceptible to developing PCOS. It should be noted that the *Fok*I polymorphism does not risk PCOS. Moreover, several limitations have been noted regarding region-specific studies, a lack of global representation, and a number of subjects in primary studies. Clearly, confounding factors like serum levels of vitamin D, dietary habits, exposure to sunlight, or body mass index may influence the activity of VDR significantly. Future study should acknowledge noted limitations, focusing on the interplay between genetics and environmental factors in the expression of PCOS. Consequently, more multicentric investigations with higher sample sizes are necessary to validate the observed associations, and genome-wide association investigations (GWAS) would significantly enhance the results.

### Ethical approval

Not applicable.

### Funding

Not applicable.

## CRediT authorship contribution statement

**Roozbeh Heidarzadehpilehrood:** Writing – review & editing, Writing – original draft, Visualization, Supervision, Software, Resources, Project administration, Methodology, Investigation, Formal analysis, Data curation. **Habibah Abdul Hamid:** Writing – review & editing, Resources, Methodology. **Maryam Pirhoushiaran:** Writing – review & editing, Writing – original draft, Supervision, Software, Project administration, Methodology, Formal analysis.

## Consent to publish

We affirm that all listed co-authors have reviewed and approved the final version of the paper and consent to its publication.

## Availability of data and materials

Data has been provided within this paper.

## Conflict of interest

The authors declared that they have no conflicts of interest in the research.

## Abbreviations List

AMH: Anti-Müllerian Hormone
CI: Confidence Interval
GCs: Granulosa Cells
HWE: Hardy–Weinberg Equilibrium
NOS: Newcastle-Ottawa Scale
OR: Odds Ratio
PCOS: Polycystic Ovary Syndrome
PRISMA: Preferred Reporting Items for Systematic Reviews and Meta-Analyses
PROSPERO: Prospective International Registry of Systematic Reviews
RCTs: Randomized Controlled Trials
SNP: Single Nucleotide Polymorphism
VDR: Vitamin D Receptor
